# Bluetongue Virus Serotype 3 and Schmallenberg Virus in *Culicoides* Biting Midges, Western Germany, 2023

**DOI:** 10.3201/eid3007.240275

**Published:** 2024-07

**Authors:** Anja Voigt, Helge Kampen, Elisa Heuser, Sophie Zeiske, Bernd Hoffmann, Dirk Höper, Mark Holsteg, Franziska Sick, Sophia Ziegler, Kerstin Wernike, Martin Beer, Doreen Werner

**Affiliations:** Leibniz-Centre for Agricultural Landscape Research, Muencheberg, Germany (A. Voigt, D. Werner);; Friedrich-Loeffler-Institut, Greifswald–Insel Riems, Germany (H. Kampen, E. Heuser, S. Zeiske, B. Hoffmann, D. Höper, F. Sick, S. Ziegler, K. Wernike, M. Beer);; Chamber of Agriculture for North Rhine-Westphalia, Bad Sassendorf, Germany (M. Holsteg)

**Keywords:** Bluetongue virus, viruses, BTV-3, vector-borne infections, Culicoides, midges, emerging diseases, livestock, cattle, sheep, Germany

## Abstract

In October 2023, bluetongue virus serotype 3 (BTV-3) emerged in Germany, where Schmallenberg virus is enzootic. We detected BTV-3 in 1 pool of *Culicoides* biting midges collected at the time ruminant infections were reported. Schmallenberg virus was found in many vector pools. Vector trapping and analysis could elucidate viral spread.

Biting midge­–borne bluetongue virus (BTV), an orbivirus of the *Sedoreoviridae* family, can cause epizootic disease in domestic and wild ruminants (*1*). Bluetongue (BT) is a World Organisation for Animal Health–listed disease and is regulated within the European Union (EU) in accordance with Regulation (EU) 2016/429 and its delegated regulations (*2*). Under those regulations, BT outbreaks require trade restrictions in EU member states to prevent the etiologic agent from spreading.

BTV serotype 3 (BTV-3) emerged in continental Europe in early September 2023, when clinical disease was observed on 4 sheep farms in the Netherlands (M. Holwerda et al., unpub. data, https://doi.org/10.1101/2023.09.29.560138). By mid-October, >1,000 outbreaks had been detected throughout the Netherlands, increasing to 5,884 by mid-December 2023 (*3*). At the same time, BTV-3 reached Belgium and was detected in the United Kingdom in November 2023 (*4*,*5*). 

In contrast to emerging BTV-3, the orthobunyavirus Schmallenberg virus (SBV) is enzootic in continental Europe; it was initially detected in 2011 near the border between Germany and the Netherlands (*6*). Another biting midge–borne virus, epizootic hemorrhagic disease virus (EHDV), emerged in Europe in 2022 (*7*). Those 3 viruses share major epidemiologic characteristics; all 3 are transmitted by *Culicoides* biting midges (Diptera: Ceratopogonidae) and affect mainly ruminants (*1*,*6*). Germany was declared free of BTV-8 in June 2023 (*8*), but on October 12, 2023, a case of BTV-3 was confirmed in a sheep in the Kleve district, close to the border with the Netherlands. By April 18, 2024, a total of 55 additional BTV-3 cases were reported from sheep and cattle farms in the federal states of North Rhine-Westphalia and Lower Saxony, Germany (Figure). We collected biting midges from those 2 states to evaluate the extent of vectorborne viruses in the region.

**Figure:**
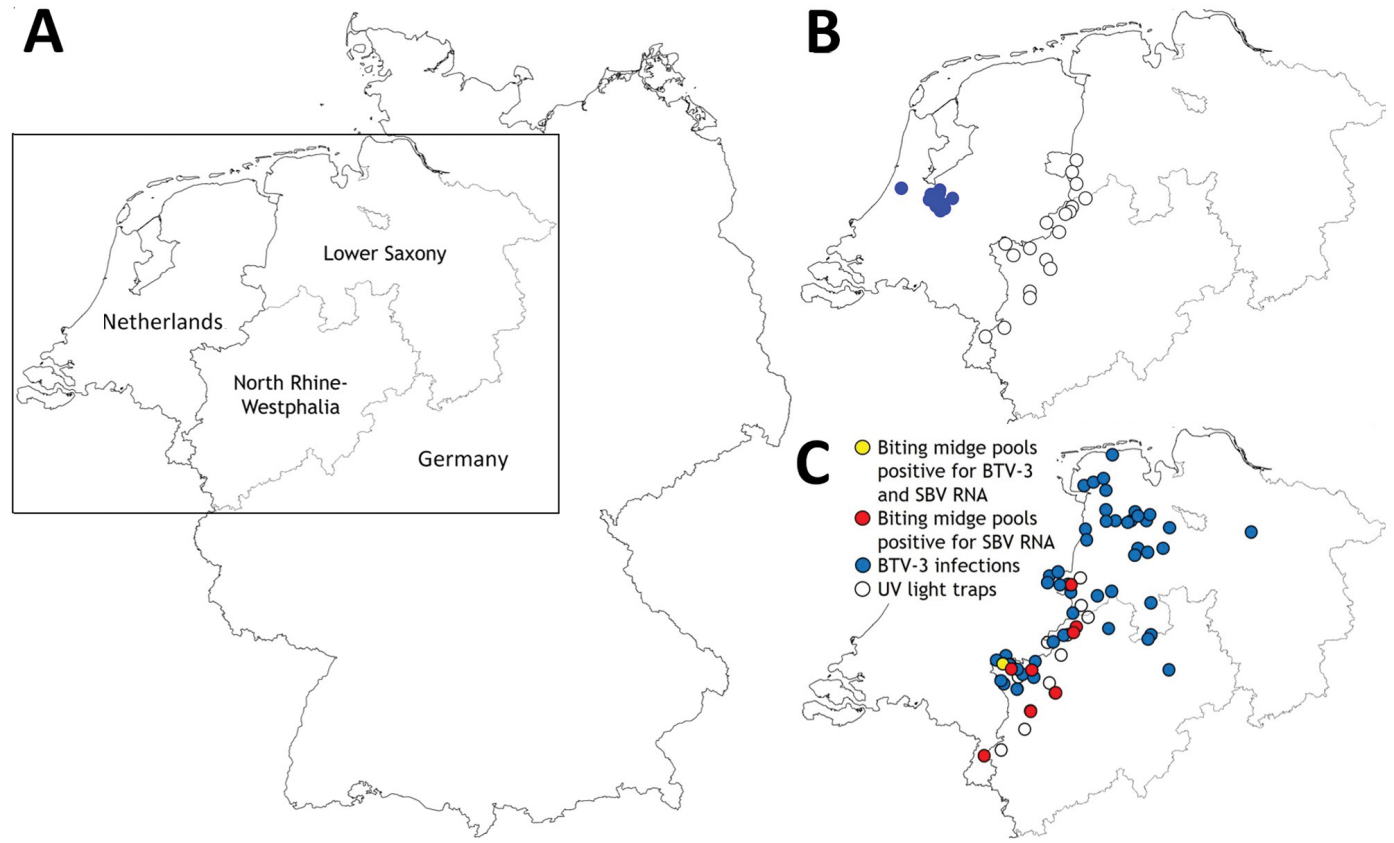
Sampling locations and infection sites in study of BTV-3 and SBV in *Culicoides* biting midges, western Germany, 2023. A) Overview map of Germany and the Netherlands showing North Rhine-Westphalia and Lower Saxony study areas. B) BTV-3 cases in the Netherlands (blue dots) as of September 8, 2023, and locations of UV light traps (white dots) along the border between Germany and the Netherlands. C) BTV-3 infections in ruminants (blue dots) reported to the animal disease reporting system in Germany as of April 18, 2024, and geographic assignment of farms with biting midge pools that tested positive for BTV-3 and SBV RNA (yellow dot) and those with pools only positive for SBV RNA (red dots). BTV-3, bluetongue virus serotype 3; SBV, Schmallenberg virus.

## The Study

After BTV-3 emerged in the Netherlands, and before any clinically suspicion cases had been announced in ruminants in Germany, we installed biting midge traps in animal stables in western Germany to collect putative BTV-3 vectors and test them for virus infection. Traps were equipped with a UV light but no CO_2_ source (Biogents, https://eu.biogents.com). During September 24–26, we set 1 trap each on 18 cattle, sheep, and goat farms in North Rhine-Westphalia and Lower Saxony, along the border with the Netherlands. We placed the traps close to the animals at sites protected from wind and rain. Traps operated continuously, and we recovered collected insects every day until November 9 or 11, depending on the location. After those dates, the traps were only activated for 24 hours per week and samples were collected that day. 

We collected biting midges, placed them in 80% ethanol and stored them at room temperature in the dark until processing. A few days, but not >4 weeks, after collection, we morphologically identified midges as *C. obsoletus* group, *C. pulicaris* complex, and other *Culicoides*. *C. obsoletus* group and *C. pulicaris* complex midges are considered the main BTV and SBV vectors in Europe (*9*). We used a multiplex quantitative reverse transcription PCR (qRT-PCR) to screen pools of <50 *C. obsoletus* group and *C. pulicaris* complexmidges for BTV and EHDV RNA (*10*). EHDV only recently emerged in southern and western Europe (*7*) and might be the next biting midge–borne virus to spread to central Europe. We also tested midge pools for SBV RNA because that virus is enzootic in the ruminant population in the region and expected to be detectable in insect vectors (*11*). We subsequently analyzed pools that tested BTV-positive by using a BTV-3–specific qRT-PCR (*12*). We retrospectively examined BTV-3–positive pools to determine the specific biting midge species (*13*,*14*).

During September 26–November 9, we collected 1,603 biting midge pools at 9 sites. The number of pools per site ranged from 27–466, depending on the number of midges collected. We tested those pools for viral RNA; 1 pool of *C. obsoletus* group midges collected in Kleve (Figure, panel C) on October 12 tested positive for BTV RNA (quantification cycle [Cq] value 35.6). We subsequently confirmed that pool as BTV-3–positive (Cq 37.5). The pool consisted of a mixture of *C. obsoletus* clade O1 (or *C. montanus*, which cannot be reliably differentiated from *C. obsolestus* clade O1 with the test system used but is not supposed to occur in Central Europe), *C. scoticus*, and *C. chiopterus*. Another pool of *C. obsoletus* group midges captured on the same day and at the same site tested SBV­ RNA–positive. In addition, we detected SBV in 534 midge pools collected during the 6.5-week period from all 9 locations: 1 site in Lower Saxony in the Grafschaft-Bentheim district, and 8 sites in North Rhine-Westphalia (3 in Kleve district, 2 in Wesel district, 2 in Borken district, and 1 in Heinsberg district) (Figure; Appendix). Except for 2 *C. pulicaris* complex pools, all SBV RNA–positive pools belonged to the *C. obsoletus* group (Appendix). No pools were positive for both BTV and SBV RNA, and all tested pools were EHDV-negative.

We calculated the minimum infection rates (MIR; i.e., number of positive pools divided by number of tested pools, multiplied by 1,000) for each virus (*15*). We found an MIR of 333.13 for SBV, indicating high circulation, and an MIR of 0.62 for BTV-3, indicating low circulation. However, MIR can be affected by pool size; the more specimens in a pool, the higher the possibility that >1 positive biting midge would be included, but that effect would not become evident when pools are examined. Conversely, the sensitivity of detection decreases with increasing pool size if only 1 positive biting midge was in the pool.

Using an isolate obtained from a BTV-3–positive sheep blood sample and further characterized on both *Culicoides* cells and baby hamster kidney cells, we produced a nearly complete genome sequence (International Nucleotide Sequence Database Collaboration, http://www.insdc.org; project no. PRJEB72862). The obtained genome was 99.94% identical to the sequence of a recent BTV-3 isolate from the Netherlands (GenBank accession nos. OR603992–4001) at the nucleotide level and 99.95% at the amino acid level. The genome segments of the strain from Germany were 83.24%–97.67% identical to the BTV-3 SAR2018 strain isolated from a sheep in Italy in 2018 (GenBank accession nos. MK348537–46) and 81.26%–97.89% identical to the TUN2016 strain isolated from a sheep in Tunisia in 2016 (GenBank accession nos. KY432369–78).

## Conclusions

BTV-3 was confirmed in an infected sheep in Germany on October 12, 2023, and viral spread was detected in 2 federal states by winter 2023–2024. The isolated BTV-3 is nearly identical to virus strains from outbreaks in the Netherlands. A pool of *C. obsoletus* group biting midges collected on a cattle farm in the same district on the same day BTV-3 was confirmed in Germany tested positive for BTV-3 RNA. Detecting BTV-3 in its putative vectors confirms an ongoing transmission cycle, albeit circulating at a low level; only 1 insect pool tested positive, and only a few animals were BTV-3–positive on affected farms. In contrast, we found SBV RNA in numerous *Culicoides* pools, reflecting its intense circulation in ruminant populations; SBV-infected cattle, sheep, and goats have been reported in Germany since 2011, although prevalence between years varies (*6*). The Cq values of the SBV qRT-PCR in some of the investigated *Culicoides* pools indicate substantial virus loads, reflecting extensive regional SBV circulation in autumn 2023 (Appendix). 

In conclusion, circulation of BTV-3 in Germany is likely to continue, intensify, and spread with the onset of seasonal biting midge activity in spring 2024. Large-scale biting midge monitoring combined with rapid analysis for viruses could contribute to an early warning system for emerging biting midge­–borne diseases.

Appendix Additional information on bluetongue virus serotype 3 and Schmallenberg virus in *Culicoides* biting midges, western Germany, 2023.

## References

[1] Maclachlan NJ, Mayo CE, Daniels PW, Savini G, Zientara S, Gibbs EP. Bluetongue. Rev Sci Tech. 2015;34:329–40. 10.20506/rst.34.2.2360 26601438

[2] European Commission. Regulation (EU) No. 2016/429 of 9 March 2016 on transmissible animal diseases and amending and repealing certain acts in the area of animal health (‘Animal Health Law’). Off J EU. 2016; L84:1–208.

[3] Leis P. Bluetongue (BTV-3). Presented: PAFF Animal Health and Welfare committee meeting; the Netherlands: December 14, 2023 [cited 24 Apr 2024]. https://food.ec.europa.eu/system/files/2023-12/reg-com_ahw_20231214123_pres-07.pdf

[4] World Animal Health Information System. Belgium—Bluetongue virus (Inf. with), event 5265, report ID 163218 [cited 2024 Apr 24]. https://wahis.woah.org/#/in-event/5265/dashboard

[5] World Animal Health Information System. United Kingdom—Bluetongue virus (Inf. with), event 5330, report ID 163824 [cited 2024 Apr 24]. https://wahis.woah.org/#/in-event/5330/dashboard

[6] Wernike K, Beer M. Schmallenberg virus: a novel virus of veterinary importance. Adv Virus Res. 2017;99:39–60. 10.1016/bs.aivir.2017.07.001 29029729

[7] Lorusso A, Cappai S, Loi F, Pinna L, Ruiu A, Puggioni G, et al. Epizootic hemorrhagic disease virus serotype 8, Italy, 2022. Emerg Infect Dis. 2023;29:1063–5. 10.3201/eid2905.221773 37081599 PMC10124640

[8] European Commission. Commission implementing regulation (EU) 2021/620. Off J EU. 2021;131:78.

[9] Mellor PS, Boorman J, Baylis M. *Culicoides* biting midges: their role as arbovirus vectors. Annu Rev Entomol. 2000;45:307–40. 10.1146/annurev.ento.45.1.307 10761580

[10] Wernike K, Hoffmann B, Beer M. Simultaneous detection of five notifiable viral diseases of cattle by single-tube multiplex real-time RT-PCR. J Virol Methods. 2015;217:28–35. 10.1016/j.jviromet.2015.02.023 25746154

[11] Bilk S, Schulze C, Fischer M, Beer M, Hlinak A, Hoffmann B. Organ distribution of Schmallenberg virus RNA in malformed newborns. Vet Microbiol. 2012;159:236–8. 10.1016/j.vetmic.2012.03.035 22516190

[12] Lorusso A, Sghaier S, Di Domenico M, Barbria ME, Zaccaria G, Megdich A, et al. Analysis of bluetongue serotype 3 spread in Tunisia and discovery of a novel strain related to the bluetongue virus isolated from a commercial sheep pox vaccine. Infect Genet Evol. 2018;59:63–71. 10.1016/j.meegid.2018.01.025 29386141

[13] Dähn O, Werner D, Mathieu B, Kampen H. Development of conventional multiplex PCR assays for the identification of 21 West Palaearctic biting midge taxa (Diptera: Ceratopogonidae) belonging to the *Culicoides* subgenus *Culicoides*, including recently discovered species and genetic variants. Diversity (Basel). 2023;15:699. 10.3390/d15060699

[14] Dähn O, Werner D, Mathieu B, Kampen H. Large-scale cytochrome c oxidase subunit I gene data analysis for the development of a multiplex polymerase chain reaction test capable of identifying biting midge vector species and haplotypes (Diptera: Ceratopogonidae) of the *Culicoides* subgenus *Avaritia* Fox, 1955. Genes (Basel). 2024;15:323. 10.3390/genes15030323 38540382 PMC10969821

[15] Walter SD, Hildreth SW, Beaty BJ. Estimation of infection rates in population of organisms using pools of variable size. Am J Epidemiol. 1980;112:124–8. 10.1093/oxfordjournals.aje.a112961 7395846

