# Supplementary material for: Bluetongue Virus Serotype 3 and Schmallenberg Virus in Culicoides Biting Midges, Western Germany, 2023
Source: Emerg Infect Dis. 2024 Jul;30(7):1438–41. doi: 10.3201/eid3007.240275 (PMC11210651; doi:10.3201/eid3007.240275)
Supplement: Appendix — Additional information on bluetongue virus serotype 3 and Schmallenberg virus in Culicoides biting midges, western Germany, 2023. [file 24-0275-Techapp-s1.pdf]

Article DOI: https://doi.org/10.3201/3007.240275

EID cannot ensure accessibility for supplementary materials supplied by authors. Readers who have difficulty accessing supplementary content should contact the authors for assistance.

Bluetongue Virus Serotype 3 and Schmallenberg Virus in *Culicoides* Biting Midges, Western Germany, 2023

Appendix

Appendix Table. Characteristics of insect pools collected in a study of bluetongue virus serotype 3 and Schmallenberg virus in *Culicoides* biting midges, Western Germany, 2023*

Pool size, no. of biting midges	Collection date	Place of collection	Type of livestock	Virus	Cq value	Biting midge group/complex	Biting midge taxon
39	12 Oct 2023	Kleve, North Rhine-Westphalia	cattle	BTV-3	35.6	Obsoletus Group	*C. obsoletus clade O1/C. montanus*<br>*C. scoticus*<br>*C. chiopterus*
50	13 Oct 2023	Nordhorn, Lower Saxony	cattle	SBV	25.6	Obsoletus Group	*C. obsoletus clade O1/C. montanus*<br>*C. scoticus*<br>*C. chiopterus*
50	13 Oct 2023	Nordhorn, Lower Saxony	cattle	SBV	30.2	Obsoletus Group	*C. obsoletus clade O1/C. montanus*<br>*C. scoticus*
50	13 Oct 2023	Nordhorn, Lower Saxony	cattle	SBV	31.6	Obsoletus Group	*C. obsoletus clade O1/C. montanus*<br>*C. scoticus*<br>*C. dewulfi*
50	13 Oct 2023	Nordhorn, Lower Saxony	cattle	SBV	25.3	Obsoletus Group	*C. obsoletus clade O1/C. montanus*<br>*C. scoticus*<br>*C. dewulfi*<br>*C. chiopterus*
50	14 Oct 2023	Nordhorn, Lower Saxony	cattle	SBV	29.6	Obsoletus Group	*C. obsoletus clade O1/C. montanus*<br>*C. scoticus*<br>*C. dewulfi*<br>*C. chiopterus*
50	12 Oct 2023	Kleve, North Rhine-Westphalia	cattle	SBV	26.3	Obsoletus Group	*C. obsoletus clade O1/C. montanus*<br>*C. scoticus*<br>*C. dewulfi*<br>*C. chiopterus*
49	12 Oct 2023	Kalkar, North Rhine-Westphalia	cattle	SBV	31.3	Obsoletus Group	*C. obsoletus clade O1/C. montanus*<br>*C. scoticus*<br>*C. chiopterus*
50	12 Oct 2023	Kalkar, North Rhine-Westphalia	cattle	SBV	25.5	Obsoletus Group	*C. obsoletus clade O1/C. montanus*<br>*C. scoticus*<br>*C. chiopterus*
50	12 Oct 2023	Kalkar, North Rhine-Westphalia	cattle	SBV	22.6	Obsoletus Group	*C. obsoletus clade O1/C. montanus*<br>*C. scoticus*

Pool size, no. of biting midges	Collection date	Place of collection	Type of livestock	Virus	Cq value	Biting midge group/complex	Biting midge taxon
							C. dewulfi
10	11 Oct 2023	Kalkar, North Rhine-Westphalia	cattle	SBV	40.0	Obsoletus Group	*C. obsoletus clade O1/C. montanus*
50	10 Oct 2023	Kalkar, North Rhine-Westphalia	cattle	SBV	35.8	Obsoletus Group	*C. obsoletus clade O1/C. montanus*<br>*C. scoticus*<br>*C. dewulfi*<br>*C. chiopterus*
50	10 Oct 2023	Kalkar, North Rhine-Westphalia	cattle	SBV	41.2	Obsoletus Group	*C. obsoletus clade O1/C. montanus*<br>*C. scoticus*<br>*C. chiopterus*
50	10 Oct 2023	Kalkar, North Rhine-Westphalia	cattle	SBV	32.4	Obsoletus Group	*C. obsoletus clade O1/C. montanus*<br>*C. scoticus*<br>*C. chiopterus*
50	10 Oct 2023	Kalkar, North Rhine-Westphalia	cattle	SBV	39.5	Obsoletus Group	*C. obsoletus clade O1/C. montanus*<br>*C. scoticus*<br>*C. dewulfi*
50	10 Oct 2023	Kalkar, North Rhine-Westphalia	cattle	SBV	36.9	Obsoletus Group	*C. obsoletus clade O1/C. montanus*<br>*C. scoticus*<br>*C. dewulfi*
50	10 Oct 2023	Kalkar, North Rhine-Westphalia	cattle	SBV	39.8	Obsoletus Group	*C. obsoletus clade O1/C. montanus*<br>*C. scoticus*
50	10 Oct 2023	Kalkar, North Rhine-Westphalia	cattle	SBV	37.9	Obsoletus Group	*C. obsoletus clade O1/C. montanus*<br>*C. scoticus*<br>*C. dewulfi*
50	10 Oct 2023	Kalkar, North Rhine-Westphalia	cattle	SBV	37.8	Obsoletus Group	*C. obsoletus clade O1/C. montanus*<br>*C. scoticus*<br>*C. dewulfi*<br>*C. chiopterus*
50	10 Oct 2023	Kalkar, North Rhine-Westphalia	cattle	SBV	38.0	Obsoletus Group	*C. obsoletus clade O1/C. montanus*<br>*C. scoticus*<br>*C. dewulfi*<br>*C. chiopterus*
50	10 Oct 2023	Kalkar, North Rhine-Westphalia	cattle	SBV	31.3	Obsoletus Group	*C. obsoletus clade O1/C. montanus*<br>*C. scoticus*<br>*C. chiopterus*
50	10 Oct 2023	Kalkar, North Rhine-Westphalia	cattle	SBV	37.5	Obsoletus Group	*C. obsoletus clade O1/C. montanus*<br>*C. scoticus*
50	09 Oct 2023	Kalkar, North Rhine-Westphalia	cattle	SBV	33.9	Obsoletus Group	*C. obsoletus clade O1/C. montanus*<br>*C. scoticus*<br>*C. dewulfi*<br>*C. chiopterus*
50	09 Oct 2023	Kalkar, North Rhine-Westphalia	cattle	SBV	31.9	Obsoletus Group	*C. obsoletus clade O1/C. montanus*<br>*C. scoticus*<br>*C. chiopterus*
50	08 Oct 2023	Kalkar, North Rhine-Westphalia	cattle	SBV	35.3	Obsoletus Group	*C. obsoletus clade O1/C. montanus*<br>*C. scoticus*<br>*C. dewulfi*<br>*C. chiopterus*
50	12 Oct 2023	Rees, North Rhine-Westphalia	cattle	SBV	23.7	Obsoletus Group	*C. obsoletus clade O1/C. montanus*

Pool size, no. of biting midges	Collection date	Place of collection	Type of livestock	Virus	Cq value	Biting midge group/complex	Biting midge taxon
							C. scoticus<br>*C. dewulfi*
17	12 Oct 2023	Rees, North Rhine-Westphalia	cattle	SBV	28.6	Obsoletus Group	*C. obsoletus clade O1/C. montanus*<br>*C. scoticus*<br>*C. dewulfi*<br>*C. chiopterus*
50	11 Oct 2023	Rees, North Rhine-Westphalia	cattle	SBV	29.0	Obsoletus Group	*C. obsoletus clade O1/C. montanus*<br>*C. scoticus*<br>*C. dewulfi*
50	11 Oct 2023	Rees, North Rhine-Westphalia	cattle	SBV	23.3	Obsoletus Group	*C. obsoletus clade O1/C. montanus*<br>*C. scoticus*<br>*C. dewulfi*<br>*C. chiopterus*
50	11 Oct 2023	Rees, North Rhine-Westphalia	cattle	SBV	24.2	Obsoletus Group	*C. obsoletus clade O1/C. montanus*<br>*C. scoticus*<br>*C. chiopterus*
50	11 Oct 2023	Rees, North Rhine-Westphalia	cattle	SBV	24.3	Obsoletus Group	*C. obsoletus clade O1/C. montanus*<br>*C. scoticus*<br>*C. dewulfi*<br>*C. chiopterus*
50	14 Oct 2023	Nordhorn, Lower Saxony	cattle	SBV	24.4	Obsoletus Group	*C. obsoletus clade O1/C. montanus*<br>*C. scoticus*<br>*C. dewulfi*
50	14 Oct 2023	Nordhorn, Lower Saxony	cattle	SBV	28.2	Obsoletus Group	*C. obsoletus clade O1/C. montanus*<br>*C. scoticus*<br>*C. dewulfi*
50	14 Oct 2023	Nordhorn, Lower Saxony	cattle	SBV	29.0	Obsoletus Group	*C. obsoletus clade O1/C. montanus*<br>*C. scoticus*<br>*C. dewulfi*
50	14 Oct 2023	Nordhorn, Lower Saxony	cattle	SBV	28.0	Obsoletus Group	*C. obsoletus clade O1/C. montanus*<br>*C. scoticus*
50	14 Oct 2023	Nordhorn, Lower Saxony	cattle	SBV	24.8	Obsoletus Group	*C. obsoletus clade O1/C. montanus*<br>*C. scoticus*<br>*C. dewulfi*<br>*C. chiopterus*
10	14 Oct 2023	Nordhorn, Lower Saxony	cattle	SBV	27.2	Pulicaris Complex	*C. punctatus*
50	09 Oct 2023	Rees, North Rhine-Westphalia	cattle	SBV	22.7	Obsoletus Group	*C. obsoletus clade O1/C. montanus*<br>*C. scoticus*<br>*C. dewulfi*<br>*C. chiopterus*
50	09 Oct 2023	Rees, North Rhine-Westphalia	cattle	SBV	25.8	Obsoletus Group	*C. obsoletus clade O1/C. montanus*<br>*C. scoticus*<br>*C. dewulfi*<br>*C. chiopterus*
50	09 Oct 2023	Rees, North Rhine-Westphalia	cattle	SBV	34.9	Obsoletus Group	*C. obsoletus clade O1/C. montanus*<br>*C. scoticus*<br>*C. dewulfi*
50	08 Oct 2023	Rees, North Rhine-Westphalia	cattle	SBV	22.5	Obsoletus Group	*C. obsoletus clade O1/C. montanus*<br>*C. scoticus*

Pool size, no. of biting midges	Collection date	Place of collection	Type of livestock	Virus	Cq value	Biting midge group/complex	Biting midge taxon
							C. dewulfi *C. chiopterus*
1	08 Oct 2023	Rees, North Rhine-Westphalia	cattle	SBV	39.2	Pulicaris Complex	*C. punctatus*
50	07 Oct 2023	Rees, North Rhine-Westphalia	cattle	SBV	27.3	Obsoletus Group	*C. obsoletus clade O1/C. montanus* *C. scoticus* *C. dewulfi* *C. chiopterus*
43	07 Oct 2023	Rees, North Rhine-Westphalia	cattle	SBV	28.3	Obsoletus Group	*C. obsoletus clade O1/C. montanus* *C. scoticus* *C. dewulfi* *C. chiopterus*
50	06 Oct 2023	Rees, North Rhine-Westphalia	cattle	SBV	24.5	Obsoletus Group	*C. obsoletus clade O1/C. montanus* *C. scoticus* *C. dewulfi* *C. chiopterus*
50	07 Oct 2023	Kalkar, North Rhine-Westphalia	cattle	SBV	36.8	Obsoletus Group	*C. obsoletus clade O1/C. montanus* *C. scoticus* *C. dewulfi*
50	07 Oct 2023	Kalkar, North Rhine-Westphalia	cattle	SBV	38.2	Obsoletus Group	*C. obsoletus clade O1/C. montanus* *C. scoticus* *C. dewulfi*
50	07 Oct 2023	Kalkar, North Rhine-Westphalia	cattle	SBV	40.1	Obsoletus Group	*C. obsoletus clade O1/C. montanus* *C. scoticus* *C. dewulfi* *C. chiopterus*
50	07 Oct 2023	Kalkar, North Rhine-Westphalia	cattle	SBV	39.8	Obsoletus Group	*C. obsoletus clade O1/C. montanus* *C. scoticus* *C. dewulfi* *C. chiopterus*
50	07 Oct 2023	Kalkar, North Rhine-Westphalia	cattle	SBV	39.0	Obsoletus Group	*C. obsoletus clade O1/C. montanus* *C. scoticus* *C. dewulfi* *C. chiopterus*
50	07 Oct 2023	Kalkar, North Rhine-Westphalia	cattle	SBV	37.2	Obsoletus Group	*C. obsoletus clade O1/C. montanus* *C. scoticus* *C. dewulfi* *C. chiopterus*
50	07 Oct 2023	Kalkar, North Rhine-Westphalia	cattle	SBV	39.8	Obsoletus Group	*C. obsoletus clade O1/C. montanus* *C. scoticus* *C. dewulfi* *C. chiopterus*
50	07 Oct 2023	Kalkar, North Rhine-Westphalia	cattle	SBV	37.3	Obsoletus Group	*C. obsoletus clade O1/C. montanus* *C. scoticus* *C. dewulfi* *C. chiopterus*
50	07 Oct 2023	Kalkar, North Rhine-Westphalia	cattle	SBV	37.8	Obsoletus Group	*C. obsoletus clade O1/C. montanus* *C. scoticus* *C. chiopterus*
50	07 Oct 2023	Kalkar, North Rhine-Westphalia	cattle	SBV	26.1	Obsoletus Group	*C. obsoletus clade O1/C. montanus* *C. scoticus*

Pool size, no. of biting midges	Collection date	Place of collection	Type of livestock	Virus	Cq value	Biting midge group/complex	Biting midge taxon
							C. dewulfi<br>*C. chiopterus*
50	07 Oct 2023	Kalkar, North Rhine-Westphalia	cattle	SBV	37.3	Obsoletus Group	*C. obsoletus clade O1/C. montanus*<br>*C. scoticus*<br>*C. chiopterus*
50	07 Oct 2023	Kalkar, North Rhine-Westphalia	cattle	SBV	41.6	Obsoletus Group	*C. obsoletus clade O1/C. montanus*<br>*C. scoticus*<br>*C. chiopterus*
50	07 Oct 2023	Kalkar, North Rhine-Westphalia	cattle	SBV	37.7	Obsoletus Group	*C. obsoletus clade O1/C. montanus*<br>*C. scoticus*<br>*C. dewulfi*<br>*C. chiopterus*
50	07 Oct 2023	Kalkar, North Rhine-Westphalia	cattle	SBV	41.6	Obsoletus Group	*C. obsoletus clade O1/C. montanus*<br>*C. scoticus*<br>*C. dewulfi*<br>*C. chiopterus*
50	07 Oct 2023	Kalkar, North Rhine-Westphalia	cattle	SBV	40.6	Obsoletus Group	*C. obsoletus clade O1/C. montanus*<br>*C. scoticus*<br>*C. dewulfi*
50	07 Oct 2023	Kalkar, North Rhine-Westphalia	cattle	SBV	41.7	Obsoletus Group	*C. obsoletus clade O1/C. montanus*<br>*C. scoticus*<br>*C. dewulfi*<br>*C. chiopterus*
50	07 Oct 2023	Kalkar, North Rhine-Westphalia	cattle	SBV	40.0	Obsoletus Group	*C. obsoletus clade O1/C. montanus*<br>*C. scoticus*<br>*C. dewulfi*<br>*C. chiopterus*
44	07 Oct 2023	Kalkar, North Rhine-Westphalia	cattle	SBV	39.2	Obsoletus Group	*C. obsoletus clade O1/C. montanus*<br>*C. scoticus*<br>*C. dewulfi*<br>*C. chiopterus*
44	07 Oct 2023	Kalkar, North Rhine-Westphalia	cattle	SBV	25.1	Obsoletus Group	*C. obsoletus clade O1/C. montanus*<br>*C. scoticus*<br>*C. dewulfi*<br>*C. chiopterus*
50	05 Oct 2023	Rees, North Rhine-Westphalia	cattle	SBV	32.4	Obsoletus Group	*C. obsoletus clade O1/C. montanus*<br>*C. scoticus*<br>*C. dewulfi*
38	7 Oct 2023	Neukirchen, North Rhine-Westphalia	goat	SBV	24.1	Obsoletus Group	*C. obsoletus clade O1/C. montanus*<br>*C. scoticus*<br>*C. dewulfi*
50	08 Oct 2023	Neukirchen, North Rhine-Westphalia	goat	SBV	30.6	Obsoletus Group	*C. obsoletus clade O1/C. montanus*<br>*C. scoticus*<br>*C. dewulfi*
50	08 Oct 2023	Neukirchen, North Rhine-Westphalia	goat	SBV	31.3	Obsoletus Group	*C. obsoletus clade O1/C. montanus*<br>*C. scoticus*<br>*C. dewulfi*
50	08 Oct 2023	Neukirchen, North Rhine-Westphalia	goat	SBV	32.0	Obsoletus Group	*C. obsoletus clade O1/C. montanus*<br>*C. scoticus*<br>*C. dewulfi*

Pool size, no. of biting midges	Collection date	Place of collection	Type of livestock	Virus	Cq value	Biting midge group/complex	Biting midge taxon
50	08 Oct 2023	Neukirchen, North Rhine-Westphalia	goat	SBV	31.5	Obsoletus Group	*C. obsoletus clade O1/C. montanus* *C. scoticus* *C. dewulfi*
50	08 Oct 2023	Neukirchen, North Rhine-Westphalia	goat	SBV	45504	Obsoletus Group	*C. obsoletus cladeO1* *C. scoticus* *C. dewulfi*
50	08 Oct 2023	Neukirchen, North Rhine-Westphalia	goat	SBV	32.2	Obsoletus Group	*C. obsoletus clade O1* *C. scoticus* *C. dewulfi*
50	08 Oct 2023	Neukirchen, North Rhine-Westphalia	goat	SBV	31.4	Obsoletus Group	*C. obsoletus clade O1/C. montanus* *C. scoticus* *C. dewulfi*
50	08 Oct 2023	Neukirchen, North Rhine-Westphalia	goat	SBV	31.5	Obsoletus Group	*C. obsoletus clade O1/C. montanus* *C. scoticus* *C. dewulfi*
50	08 Oct 2023	Neukirchen, North Rhine-Westphalia	goat	SBV	32.0	Obsoletus Group	*C. obsoletus clade O1/C. montanus* *C. scoticus* *C. dewulfi*
50	08 Oct 2023	Neukirchen, North Rhine-Westphalia	goat	SBV	31.3	Obsoletus Group	*C. obsoletus clade O1/C. montanus* *C. scoticus* *C. dewulfi*
50	08 Oct 2023	Neukirchen, North Rhine-Westphalia	goat	SBV	31.1	Obsoletus Group	*C. obsoletus clade O1/C. montanus* *C. scoticus* *C. dewulfi*
50	08 Oct 2023	Neukirchen, North Rhine-Westphalia	goat	SBV	31.2	Obsoletus Group	*C. obsoletus clade O1/C. montanus* *C. scoticus* *C. dewulfi*
50	08 Oct 2023	Neukirchen, North Rhine-Westphalia	goat	SBV	32.0	Obsoletus Group	*C. obsoletus clade O1/C. montanus* *C. scoticus* *C. dewulfi*
50	08 Oct 2023	Neukirchen, North Rhine-Westphalia	goat	SBV	32.0	Obsoletus Group	*C. obsoletus clade O1/C. montanus* *C. scoticus* *C. dewulfi*
50	08 Oct 2023	Neukirchen, North Rhine-Westphalia	goat	SBV	32.7	Obsoletus Group	*C. obsoletus clade O1/C. montanus* *C. scoticus* *C. dewulfi*
50	08 Oct 2023	Neukirchen, North Rhine-Westphalia	goat	SBV	32.0	Obsoletus Group	*C. obsoletus clade O1/C. montanus* *C. scoticus* *C. dewulfi*
50	08 Oct 2023	Neukirchen, North Rhine-Westphalia	goat	SBV	31.3	Obsoletus Group	*C. obsoletus clade O1/C. montanus* *C. scoticus* *C. dewulfi*
50	08 Oct 2023	Neukirchen, North Rhine-Westphalia	goat	SBV	31.7	Obsoletus Group	*C. obsoletus clade O1/C. montanus* *C. scoticus* *C. dewulfi*
50	08 Oct 2023	Neukirchen, North Rhine-Westphalia	goat	SBV	31.5	Obsoletus Group	*C. obsoletus clade O1/C. montanus* *C. scoticus* *C. dewulfi*
50	08 Oct 2023	Neukirchen, North Rhine-Westphalia	goat	SBV	24.7	Obsoletus Group	*C. obsoletus clade O1/C. montanus*

Pool size, no. of biting midges	Collection date	Place of collection	Type of livestock	Virus	Cq value	Biting midge group/complex	Biting midge taxon
21	08 Oct 2023	Neukirchen, North Rhine-Westphalia	goat	SBV	33.7	Obsoletus Group	*C. scoticus*<br>*C. dewulfi*<br>*C. obsoletus clade O1/C. montanus*
44	08 Oct 2023	Neukirchen, North Rhine-Westphalia	goat	SBV	36.9	Obsoletus Group	*C. scoticus*<br>*C. dewulfi*<br>*C. obsoletus clade O1/C. montanus*
50	12 Oct 2023	Neukirchen, North Rhine-Westphalia	goat	SBV	28.7	Obsoletus Group	*C. scoticus*<br>*C. dewulfi*<br>*C. obsoletus clade O1/C. montanus*
50	01 Oct 2023	Kalkar, North Rhine-Westphalia	cattle	SBV	37.2	Obsoletus Group	*C. scoticus*<br>*C. dewulfi*<br>*C. obsoletus clade O1/C. montanus*
50	01 Oct 2023	Kalkar, North Rhine-Westphalia	cattle	SBV	36.8	Obsoletus Group	*C. scoticus*<br>*C. dewulfi*<br>*C. obsoletus clade O1/C. montanus*
50	01 Oct 2023	Kalkar, North Rhine-Westphalia	cattle	SBV	25.5	Obsoletus Group	*C. scoticus*<br>*C. chiopterus*<br>*C. obsoletus clade O1/C. montanus*
50	01 Oct 2023	Kalkar, North Rhine-Westphalia	cattle	SBV	39.6	Obsoletus Group	*C. scoticus*<br>*C. chiopterus*<br>*C. obsoletus clade O1/C. montanus*
50	01 Oct 2023	Kalkar, North Rhine-Westphalia	cattle	SBV	37.6	Obsoletus Group	*C. scoticus*<br>*C. dewulfi*<br>*C. chiopterus*<br>*C. obsoletus clade O1/C. montanus*
50	01 Oct 2023	Kalkar, North Rhine-Westphalia	cattle	SBV	37.2	Obsoletus Group	*C. scoticus*<br>*C. chiopterus*<br>*C. obsoletus clade O1/C. montanus*
50	01 Oct 2023	Kalkar, North Rhine-Westphalia	cattle	SBV	37.2	Obsoletus Group	*C. scoticus*<br>*C. dewulfi*<br>*C. obsoletus clade O1/C. montanus.*
50	01 Oct 2023	Kalkar, North Rhine-Westphalia	cattle	SBV	35.7	Obsoletus Group	*C. scoticus*<br>*C. dewulfi*<br>*C. chiopterus*<br>*C. obsoletus clade O1/C. montanus*
50	01 Oct 2023	Kalkar, North Rhine-Westphalia	cattle	SBV	37.1	Obsoletus Group	*C. scoticus*<br>*C. dewulfi*<br>*C. obsoletus clade O1/C. montanus*
50	01 Oct 2023	Kalkar, North Rhine-Westphalia	cattle	SBV	36.5	Obsoletus Group	*C. scoticus*<br>*C. dewulfi*<br>*C. obsoletus clade O1/C. montanus*
50	01 Oct 2023	Kalkar, North Rhine-Westphalia	cattle	SBV	41.2	Obsoletus Group	*C. scoticus*<br>*C. dewulfi*<br>*C. obsoletus clade O1/C. montanus*
50	01 Oct 2023	Kalkar, North Rhine-Westphalia	cattle	SBV	37.3	Obsoletus Group	*C. scoticus*<br>*C. dewulfi*<br>*C. obsoletus clade O1/C. montanus*
50	01 Oct 2023	Kalkar, North Rhine-Westphalia	cattle	SBV	38.4	Obsoletus Group	*C. scoticus*<br>*C. obsoletus clade O1/C. montanus*

Pool size, no. of biting midges	Collection date	Place of collection	Type of livestock	Virus	Cq value	Biting midge group/complex	Biting midge taxon
							C. scoticus
50	01 Oct 2023	Kalkar, North Rhine-Westphalia	cattle	SBV	35.0	Obsoletus Group	*C. obsoletus clade O1/C. montanus* *C. scoticus*
50	01 Oct 2023	Kalkar, North Rhine-Westphalia	cattle	SBV	36.8	Obsoletus Group	*C. obsoletus clade O1/C. montanus* *C. scoticus* *C. dewulfi*
50	01 Oct 2023	Kalkar, North Rhine-Westphalia	cattle	SBV	28.6	Obsoletus Group	*C. obsoletus clade O1/C. montanus* *C. scoticus* *C. dewulfi*
50	01 Oct 2023	Kalkar, North Rhine-Westphalia	cattle	SBV	36.8	Obsoletus Group	*C. obsoletus clade O1/C. montanus* *C. scoticus*
50	01 Oct 2023	Kalkar, North Rhine-Westphalia	cattle	SBV	37.0	Obsoletus Group	*C. obsoletus clade O1/C. montanus* *C. scoticus* *C. dewulfi*
50	01 Oct 2023	Kalkar, North Rhine-Westphalia	cattle	SBV	35.8	Obsoletus Group	*C. obsoletus clade O1/C. montanus* *C. scoticus* *C. chiopterus*
50	01 Oct 2023	Kalkar, North Rhine-Westphalia	cattle	SBV	35.8	Obsoletus Group	*C. obsoletus clade O1/C. montanus* *C. scoticus* *C. dewulfi* *C. chiopterus*
50	01 Oct 2023	Kalkar, North Rhine-Westphalia	cattle	SBV	27.2	Obsoletus Group	*C. obsoletus clade O1/C. montanus* *C. scoticus* *C. dewulfi*
47	01 Oct 2023	Kalkar, North Rhine-Westphalia	cattle	SBV	36.0	Obsoletus Group	*C. obsoletus clade O1/C. montanus* *C. scoticus* *C. dewulfi*
50	02 Oct 2023	Kalkar, North Rhine-Westphalia	cattle	SBV	36.4	Obsoletus Group	*C. obsoletus clade O1/C. montanus* *C. scoticus* *C. dewulfi* *C. chiopterus*
50	02 Oct 2023	Kalkar, North Rhine-Westphalia	cattle	SBV	36.5	Obsoletus Group	*C. obsoletus clade O1/C. montanus* *C. scoticus* *C. dewulfi* *C. chiopterus*
50	02 Oct 2023	Kalkar, North Rhine-Westphalia	cattle	SBV	38.5	Obsoletus Group	*C. obsoletus clade O1/C. montanus* *C. scoticus* *C. dewulfi* *C. chiopterus*
50	02 Oct 2023	Kalkar, North Rhine-Westphalia	cattle	SBV	37.8	Obsoletus Group	*C. obsoletus clade O1/C. montanus* *C. scoticus* *C. dewulfi* *C. chiopterus*
50	02 Oct 2023	Kalkar, North Rhine-Westphalia	cattle	SBV	27.6	Obsoletus Group	*C. obsoletus clade O1/C. montanus* *C. scoticus* *C. dewulfi* *C. chiopterus*
50	02 Oct 2023	Kalkar, North Rhine-Westphalia	cattle	SBV	36.2	Obsoletus Group	*C. obsoletus clade O1/C. montanus* *C. scoticus* *C. dewulfi*

Pool size, no. of biting midges	Collection date	Place of collection	Type of livestock	Virus	Cq value	Biting midge group/complex	Biting midge taxon
49	02 Oct 2023	Kalkar, North Rhine-Westphalia	cattle	SBV	35.9	Obsoletus Group	*C. chiopterus*<br>*C. obsoletus clade O1/C. montanus*<br>*C. scoticus*<br>*C. dewulfi*
50	03 Oct 2023	Kalkar, North Rhine-Westphalia	cattle	SBV	38.4	Obsoletus Group	*C. chiopterus*<br>*C. obsoletus clade O1/C. montanus*<br>*C. scoticus*<br>*C. dewulfi*
50	03 Oct 2023	Kalkar, North Rhine-Westphalia	cattle	SBV	38.5	Obsoletus Group	*C. chiopterus*<br>*C. obsoletus clade O1/C. montanus*<br>*C. scoticus*<br>*C. dewulfi*
50	03 Oct 2023	Kalkar, North Rhine-Westphalia	cattle	SBV	38.2	Obsoletus Group	*C. chiopterus*<br>*C. obsoletus clade O1/C. montanus*<br>*C. scoticus*<br>*C. dewulfi*
50	03 Oct 2023	Kalkar, North Rhine-Westphalia	cattle	SBV	37.5	Obsoletus Group	*C. chiopterus*<br>*C. obsoletus clade O1/C. montanus*<br>*C. scoticus*<br>*C. dewulfi*
50	03 Oct 2023	Kalkar, North Rhine-Westphalia	cattle	SBV	27.9	Obsoletus Group	*C. chiopterus*<br>*C. obsoletus clade O1/C. montanus*<br>*C. scoticus*<br>*C. dewulfi*
50	03 Oct 2023	Kalkar, North Rhine-Westphalia	cattle	SBV	27.9	Obsoletus Group	*C. chiopterus*<br>*C. obsoletus clade O1/C. montanus*<br>*C. scoticus*<br>*C. dewulfi*
50	03 Oct 2023	Kalkar, North Rhine-Westphalia	cattle	SBV	38.9	Obsoletus Group	*C. chiopterus*<br>*C. obsoletus clade O1/C. montanus*<br>*C. scoticus*<br>*C. dewulfi*
50	03 Oct 2023	Kalkar, North Rhine-Westphalia	cattle	SBV	45351	Obsoletus Group	*C. chiopterus*<br>*C. obsoletus clade O1/C. montanus*<br>*C. scoticus*<br>*C. dewulfi*
39	03 Oct 2023	Kalkar, North Rhine-Westphalia	cattle	SBV	38.3	Obsoletus Group	*C. obsoletus clade O1/C. montanus*<br>*C. scoticus*<br>*C. dewulfi*
50	04 Oct 2023	Kalkar, North Rhine-Westphalia	cattle	SBV	38.8	Obsoletus Group	*C. obsoletus clade O1/C. montanus*<br>*C. scoticus*<br>*C. dewulfi*
50	04 Oct 2023	Kalkar, North Rhine-Westphalia	cattle	SBV	29.5	Obsoletus Group	*C. obsoletus clade O1/C. montanus*<br>*C. scoticus*<br>*C. dewulfi*
50	04 Oct 2023	Kalkar, North Rhine-Westphalia	cattle	SBV	38.4	Obsoletus Group	*C. obsoletus clade O1/C. montanus*<br>*C. scoticus*<br>*C. dewulfi*
50	04 Oct 2023	Kalkar, North Rhine-Westphalia	cattle	SBV	38.4	Obsoletus Group	*C. chiopterus*<br>*C. obsoletus clade O1/C. montanus*<br>*C. scoticus*

Pool size, no. of biting midges	Collection date	Place of collection	Type of livestock	Virus	Cq value	Biting midge group/complex	Biting midge taxon
							C. dewulfi<br>*C. chiopterus*
50	04 Oct 2023	Kalkar, North Rhine-Westphalia	cattle	SBV	37.4	Obsoletus Group	*C. obsoletus clade O1/C. montanus*<br>*C. scoticus*<br>*C. dewulfi*
50	04 Oct 2023	Kalkar, North Rhine-Westphalia	cattle	SBV	31.4	Obsoletus Group	*C. obsoletus clade O1/C. montanus*<br>*C. scoticus*<br>*C. dewulfi*<br>*C. chiopterus*
50	04 Oct 2023	Kalkar, North Rhine-Westphalia	cattle	SBV	37..1	Obsoletus Group	*C. obsoletus clade O1/C. montanus*<br>*C. scoticus*<br>*C. dewulfi*
50	04 Oct 2023	Kalkar, North Rhine-Westphalia	cattle	SBV	35.6	Obsoletus Group	*C. obsoletus clade O1/C. montanus*<br>*C. scoticus*<br>*C. dewulfi*
50	05 Oct 2023	Kalkar, North Rhine-Westphalia	cattle	SBV	35.6	Obsoletus Group	*C. obsoletus clade O1/C. montanus*<br>*C. scoticus*<br>*C. dewulfi*
50	05 Oct 2023	Kalkar, North Rhine-Westphalia	cattle	SBV	31.3	Obsoletus Group	*C. obsoletus clade O1/C. montanus*<br>*C. scoticus*<br>*C. dewulfi*<br>*C. chiopterus*
50	05 Oct 2023	Kalkar, North Rhine-Westphalia	cattle	SBV	37.3	Obsoletus Group	*C. obsoletus clade O1/C. montanus*<br>*C. scoticus*<br>*C. dewulfi*
50	06 Oct 2023	Kalkar, North Rhine-Westphalia	cattle	SBV	37.4	Obsoletus Group	*C. obsoletus clade O1/C. montanus*<br>*C. scoticus*<br>*C. dewulfi*
50	06 Oct 2023	Kalkar, North Rhine-Westphalia	cattle	SBV	37.5	Obsoletus Group	*C. obsoletus clade O1/C. montanus*<br>*C. scoticus*<br>*C. dewulfi*<br>*C. chiopterus*
50	06 Oct 2023	Kalkar, North Rhine-Westphalia	cattle	SBV	27.4	Obsoletus Group	*C. obsoletus clade O1/C. montanus*<br>*C. scoticus*<br>*C. chiopterus*
50	06 Oct 2023	Kalkar, North Rhine-Westphalia	cattle	SBV	35.0	Obsoletus Group	*C. obsoletus clade O1/C. montanus*<br>*C. scoticus*<br>*C. dewulfi*<br>*C. chiopterus*
50	06 Oct 2023	Kalkar, North Rhine-Westphalia	cattle	SBV	35.1	Obsoletus Group	*C. obsoletus clade O1/C. montanus*<br>*C. scoticus*<br>*C. dewulfi*<br>*C. chiopterus*
50	06 Oct 2023	Kalkar, North Rhine-Westphalia	cattle	SBV	35.0	Obsoletus Group	*C. obsoletus clade O1/C. montanus*<br>*C. scoticus*<br>*C. dewulfi*<br>*C. chiopterus*
50	06 Oct 2023	Kalkar, North Rhine-Westphalia	cattle	SBV	33.8	Obsoletus Group	*C. obsoletus clade O1/C. montanus*<br>*C. scoticus*<br>*C. dewulfi*<br>*C. chiopterus*

Pool size, no. of biting midges	Collection date	Place of collection	Type of livestock	Virus	Cq value	Biting midge group/complex	Biting midge taxon
50	06 Oct 2023	Kalkar, North Rhine-Westphalia	cattle	SBV	34.9	Obsoletus Group	*C. obsoletus clade O1/C. montanus*<br>*C. scoticus*<br>*C. dewulfi*
50	06 Oct 2023	Kalkar, North Rhine-Westphalia	cattle	SBV	34.0	Obsoletus Group	*C. obsoletus clade O1/C. montanus*<br>*C. scoticus*<br>*C. chiopterus*
50	06 Oct 2023	Kalkar, North Rhine-Westphalia	cattle	SBV	35.0	Obsoletus Group	*C. obsoletus clade O1/C. montanus*<br>*C. scoticus*<br>*C. dewulfi*<br>*C. chiopterus*
50	06 Oct 2023	Kalkar, North Rhine-Westphalia	cattle	SBV	34.6	Obsoletus Group	*C. obsoletus clade O1/C. montanus*<br>*C. scoticus*<br>*C. chiopterus*
50	06 Oct 2023	Kalkar, North Rhine-Westphalia	cattle	SBV	34.6	Obsoletus Group	*C. obsoletus clade O1/C. montanus*<br>*C. scoticus*<br>*C. dewulfi*<br>*C. chiopterus*
50	06 Oct 2023	Kalkar, North Rhine-Westphalia	cattle	SBV	35.8	Obsoletus Group	*C. obsoletus clade O1/C. montanus*<br>*C. scoticus*<br>*C. dewulfi*<br>*C. chiopterus*
50	06 Oct 2023	Kalkar, North Rhine-Westphalia	cattle	SBV	34.3	Obsoletus Group	*C. obsoletus clade O1/C. montanus*<br>*C. scoticus*<br>*C. dewulfi*<br>*C. chiopterus*
50	06 Oct 2023	Kalkar, North Rhine-Westphalia	cattle	SBV	36.1	Obsoletus Group	*C. obsoletus clade O1/C. montanus*<br>*C. scoticus*<br>*C. dewulfi*<br>*C. chiopterus*
50	06 Oct 2023	Kalkar, North Rhine-Westphalia	cattle	SBV	35.2	Obsoletus Group	*C. obsoletus clade O1/C. montanus*<br>*C. scoticus*<br>*C. dewulfi*
50	06 Oct 2023	Kalkar, North Rhine-Westphalia	cattle	SBV	36.3	Obsoletus Group	*C. obsoletus clade O1/C. montanus*<br>*C. scoticus*<br>*C. dewulfi*<br>*C. chiopterus*
50	06 Oct 2023	Kalkar, North Rhine-Westphalia	cattle	SBV	35.1	Obsoletus Group	*C. obsoletus clade O1/C. montanus*<br>*C. scoticus*<br>*C. dewulfi*
50	06 Oct 2023	Kalkar, North Rhine-Westphalia	cattle	SBV	34.4	Obsoletus Group	*C. obsoletus clade O1/C. montanus*<br>*C. scoticus*<br>*C. dewulfi*<br>*C. chiopterus*
50	06 Oct 2023	Kalkar, North Rhine-Westphalia	cattle	SBV	34.4	Obsoletus Group	*C. obsoletus clade O1/C. montanus*<br>*C. scoticus*<br>*C. chiopterus*
50	06 Oct 2023	Kalkar, North Rhine-Westphalia	cattle	SBV	33.4	Obsoletus Group	*C. obsoletus clade O1/C. montanus*<br>*C. scoticus*<br>*C. dewulfi*<br>*C. chiopterus*

Pool size, no. of biting midges	Collection date	Place of collection	Type of livestock	Virus	Cq value	Biting midge group/complex	Biting midge taxon
50	06 Oct 2023	Kalkar, North Rhine-Westphalia	cattle	SBV	36.1	Obsoletus Group	*C. obsoletus clade O1/C. montanus*<br>*C. scoticus*<br>*C. chiopterus*
50	06 Oct 2023	Kalkar, North Rhine-Westphalia	cattle	SBV	35.1	Obsoletus Group	*C. obsoletus clade O1/C. montanus*<br>*C. scoticus*<br>*C. dewulfi*<br>*C. chiopterus*
50	06 Oct 2023	Kalkar, North Rhine-Westphalia	cattle	SBV	35.9	Obsoletus Group	*C. obsoletus clade O1/C. montanus*<br>*C. scoticus*<br>*C. chiopterus*
50	06 Oct 2023	Kalkar, North Rhine-Westphalia	cattle	SBV	35.3	Obsoletus Group	*C. obsoletus clade O1/C. montanus*<br>*C. scoticus*<br>*C. dewulfi*<br>*C. chiopterus*
50	06 Oct 2023	Kalkar, North Rhine-Westphalia	cattle	SBV	26.7	Obsoletus Group	*C. obsoletus clade O1/C. montanus*<br>*C. scoticus*<br>*C. dewulfi*
50	06 Oct 2023	Kalkar, North Rhine-Westphalia	cattle	SBV	34.4	Obsoletus Group	*C. obsoletus clade O1/C. montanus*<br>*C. scoticus*<br>*C. chiopterus*
50	06 Oct 2023	Kalkar, North Rhine-Westphalia	cattle	SBV	35.2	Obsoletus Group	*C. obsoletus clade O1/C. montanus*<br>*C. scoticus*<br>*C. chiopterus*
50	06 Oct 2023	Kalkar, North Rhine-Westphalia	cattle	SBV	35.3	Obsoletus Group	*C. obsoletus clade O1/C. montanus*<br>*C. scoticus*<br>*C. dewulfi*<br>*C. chiopterus*
50	06 Oct 2023	Kalkar, North Rhine-Westphalia	cattle	SBV	36.2	Obsoletus Group	*C. obsoletus clade O1/C. montanus*<br>*C. scoticus*<br>*C. dewulfi*<br>*C. chiopterus*
50	06 Oct 2023	Kalkar, North Rhine-Westphalia	cattle	SBV	35.1	Obsoletus Group	*C. obsoletus clade O1/C. montanus*<br>*C. scoticus*<br>*C. chiopterus*
50	06 Oct 2023	Kalkar, North Rhine-Westphalia	cattle	SBV	35.1	Obsoletus Group	*C. obsoletus clade O1/C. montanus*<br>*C. scoticus*<br>*C. dewulfi*<br>*C. chiopterus*
50	06 Oct 2023	Kalkar, North Rhine-Westphalia	cattle	SBV	34.7	Obsoletus Group	*C. obsoletus clade O1/C. montanus*<br>*C. scoticus*<br>*C. dewulfi*<br>*C. chiopterus*
50	06 Oct 2023	Kalkar, North Rhine-Westphalia	cattle	SBV	36.1	Obsoletus Group	*C. obsoletus clade O1/C. montanus*<br>*C. scoticus*<br>*C. dewulfi*<br>*C. chiopterus*
50	06 Oct 2023	Kalkar, North Rhine-Westphalia	cattle	SBV	34.3	Obsoletus Group	*C. obsoletus clade O1/C. montanus*<br>*C. scoticus*<br>*C. dewulfi*<br>*C. chiopterus*

Pool size, no. of biting midges	Collection date	Place of collection	Type of livestock	Virus	Cq value	Biting midge group/complex	Biting midge taxon
50	06 Oct 2023	Kalkar, North Rhine-Westphalia	cattle	SBV	36.2	Obsoletus Group	*C. obsoletus clade O1/C. montanus*<br>*C. scoticus*<br>*C. dewulfi*<br>*C. chiopterus*
50	06 Oct 2023	Kalkar, North Rhine-Westphalia	cattle	SBV	34.9	Obsoletus Group	*C. obsoletus clade O1/C. montanus*<br>*C. scoticus*<br>*C. chiopterus*
50	06 Oct 2023	Kalkar, North Rhine-Westphalia	cattle	SBV	34.5	Obsoletus Group	*C. obsoletus clade O1/C. montanus*<br>*C. scoticus*<br>*C. dewulfi*<br>*C. chiopterus*
50	06 Oct 2023	Kalkar, North Rhine-Westphalia	cattle	SBV	34.7	Obsoletus Group	*C. obsoletus clade O1/C. montanus*<br>*C. scoticus*<br>*C. chiopterus*
50	06 Oct 2023	Kalkar, North Rhine-Westphalia	cattle	SBV	35.4	Obsoletus Group	*C. obsoletus clade O1/C. montanus*<br>*C. scoticus*<br>*C. dewulfi*<br>*C. chiopterus*
50	06 Oct 2023	Kalkar, North Rhine-Westphalia	cattle	SBV	35.4	Obsoletus Group	*C. obsoletus clade O1/C. montanus*<br>*C. scoticus*<br>*C. dewulfi*<br>*C. chiopterus*
50	06 Oct 2023	Kalkar, North Rhine-Westphalia	cattle	SBV	34.0	Obsoletus Group	*C. obsoletus clade O1/C. montanus*<br>*C. scoticus*<br>*C. dewulfi*<br>*C. chiopterus*
50	06 Oct 2023	Kalkar, North Rhine-Westphalia	cattle	SBV	34.7	Obsoletus Group	*C. obsoletus clade O1/C. montanus*<br>*C. scoticus*<br>*C. dewulfi*<br>*C. chiopterus*
50	06 Oct 2023	Kalkar, North Rhine-Westphalia	cattle	SBV	38.3	Obsoletus Group	*C. obsoletus clade O1/C. montanus*<br>*C. scoticus*<br>*C. dewulfi*<br>*C. chiopterus*
50	06 Oct 2023	Kalkar, North Rhine-Westphalia	cattle	SBV	29.5	Obsoletus Group	*C. obsoletus clade O1/C. montanus*<br>*C. scoticus*<br>*C. dewulfi*<br>*C. chiopterus*
50	06 Oct 2023	Kalkar, North Rhine-Westphalia	cattle	SBV	35.0	Obsoletus Group	*C. obsoletus clade O1/C. montanus*<br>*C. scoticus*<br>*C. dewulfi*<br>*C. chiopterus*
50	06 Oct 2023	Kalkar, North Rhine-Westphalia	cattle	SBV	35.8	Obsoletus Group	*C. obsoletus clade O1/C. montanus*<br>*C. scoticus*<br>*C. dewulfi*<br>*C. chiopterus*
50	29 Sept 2023	Kalkar, North Rhine-Westphalia	cattle	SBV	35.7	Obsoletus Group	*C. obsoletus clade O1/C. montanus*<br>*C. scoticus*<br>*C. dewulfi*<br>*C. chiopterus*
50	29 Sept 2023	Kalkar, North Rhine-Westphalia	cattle	SBV	37.1	Obsoletus Group	*C. obsoletus clade O1/C. montanus*

Pool size, no. of biting midges	Collection date	Place of collection	Type of livestock	Virus	Cq value	Biting midge group/complex	Biting midge taxon
							C. scoticus<br>*C. dewulfi*<br>*C. chiopterus*
50	29 Sept 2023	Kalkar, North Rhine-Westphalia	cattle	SBV	38.2	Obsoletus Group	*C. obsoletus clade O1/C. montanus*<br>*C. scoticus*<br>*C. dewulfi*<br>*C. chiopterus*
50	29 Sept 2023	Kalkar, North Rhine-Westphalia	cattle	SBV	34.6	Obsoletus Group	*C. obsoletus clade O1/C. montanus*<br>*C. scoticus*<br>*C. dewulfi*<br>*C. chiopterus*
50	29 Sept 2023	Kalkar, North Rhine-Westphalia	cattle	SBV	39.9	Obsoletus Group	*C. obsoletus clade O1/C. montanus*<br>*C. dewulfi*<br>*C. chiopterus*
50	29 Sept 2023	Kalkar, North Rhine-Westphalia	cattle	SBV	36.8	Obsoletus Group	*C. obsoletus clade O1/C. montanus*<br>*C. scoticus*<br>*C. dewulfi*<br>*C. chiopterus*
50	29 Sept 2023	Kalkar, North Rhine-Westphalia	cattle	SBV	45319	Obsoletus Group	*C. obsoletus clade O1/C. montanus*<br>*C. scoticus*<br>*C. dewulfi*<br>*C. chiopterus*
50	29 Sept 2023	Kalkar, North Rhine-Westphalia	cattle	SBV	26.5	Obsoletus Group	*C. obsoletus clade O1/C. montanus*<br>*C. scoticus*<br>*C. dewulfi*<br>*C. chiopterus*
50	29 Sept 2023	Kalkar, North Rhine-Westphalia	cattle	SBV	27.6	Obsoletus Group	*C. obsoletus clade O1/C. montanus*<br>*C. scoticus*<br>*C. dewulfi*
50	29 Sept 2023	Kalkar, North Rhine-Westphalia	cattle	SBV	37.4	Obsoletus Group	*C. obsoletus clade O1/C. montanus*<br>*C. scoticus*<br>*C. chiopterus*
50	27 Sept 2023	Kalkar, North Rhine-Westphalia	cattle	SBV	40.1	Obsoletus Group	*C. obsoletus clade O1/C. montanus*<br>*C. scoticus*<br>*C. dewulfi*
50	27 Sept 2023	Kalkar, North Rhine-Westphalia	cattle	SBV	34.5	Obsoletus Group	*C. obsoletus clade O1/C. montanus*<br>*C. scoticus*<br>*C. chiopterus*
50	27 Sept 2023	Kalkar, North Rhine-Westphalia	cattle	SBV	39.5	Obsoletus Group	*C. obsoletus clade O1/C. montanus*<br>*C. scoticus*<br>*C. dewulfi*
50	27 Sept 2023	Kalkar, North Rhine-Westphalia	cattle	SBV	36.8	Obsoletus Group	*C. obsoletus clade O1/C. montanus*<br>*C. scoticus*<br>*C. dewulfi*
50	27 Sept 2023	Kalkar, North Rhine-Westphalia	cattle	SBV	39.8	Obsoletus Group	*C. obsoletus clade O1/C. montanus*<br>*C. scoticus*<br>*C. dewulfi*
50	27 Sept 2023	Kalkar, North Rhine-Westphalia	cattle	SBV	39.5	Obsoletus Group	*C. obsoletus clade O1/C. montanus*<br>*C. scoticus*<br>*C. dewulfi*

Pool size, no. of biting midges	Collection date	Place of collection	Type of livestock	Virus	Cq value	Biting midge group/complex	Biting midge taxon
50	27 Sept 2023	Kalkar, North Rhine-Westphalia	cattle	SBV	31.5	Obsoletus Group	*C. obsoletus clade O1/C. montanus* *C. scoticus* *C. dewulfi*
50	27 Sept 2023	Kalkar, North Rhine-Westphalia	cattle	SBV	38.1	Obsoletus Group	*C. obsoletus clade O1/C. montanus* *C. scoticus* *C. dewulfi*
50	27 Sept 2023	Kalkar, North Rhine-Westphalia	cattle	SBV	41.1	Obsoletus Group	*C. obsoletus clade O1/C. montanus* *C. scoticus* *C. dewulfi* *C. chiopterus*
50	27 Sept 2023	Kalkar, North Rhine-Westphalia	cattle	SBV	30.1	Obsoletus Group	*C. obsoletus clade O1/C. montanus* *C. scoticus* *C. dewulfi* *C. chiopterus*
50	27 Sept 2023	Kalkar, North Rhine-Westphalia	cattle	SBV	37.4	Obsoletus Group	*C. obsoletus clade O1/C. montanus* *C. scoticus* *C. dewulfi*
50	27 Sept 2023	Kalkar, North Rhine-Westphalia	cattle	SBV	37.7	Obsoletus Group	*C. obsoletus clade O1/C. montanus* *C. scoticus* *C. dewulfi*
50	27 Sept 2023	Kalkar, North Rhine-Westphalia	cattle	SBV	37.9	Obsoletus Group	*C. obsoletus clade O1/C. montanus* *C. scoticus* *C. dewulfi* *C. chiopterus*
50	27 Sept 2023	Kalkar, North Rhine-Westphalia	cattle	SBV	39.8	Obsoletus Group	*C. obsoletus clade O1/C. montanus* *C. scoticus* *C. dewulfi*
50	07 Oct 2023	Nordhorn, Lower Saxony	cattle	SBV	35.3	Obsoletus Group	*C. obsoletus clade O1/C. montanus* *C. scoticus* *C. dewulfi*
50	07 Oct 2023	Nordhorn, Lower Saxony	cattle	SBV	33.4	Obsoletus Group	*C. obsoletus clade O1/C. montanus* *C. scoticus* *C. dewulfi* *C. chiopterus*
50	07 Oct 2023	Nordhorn, Lower Saxony	cattle	SBV	33.5	Obsoletus Group	*C. obsoletus clade O1/C. montanus* *C. scoticus* *C. dewulfi*
50	07 Oct 2023	Nordhorn, Lower Saxony	cattle	SBV	29.5	Obsoletus Group	*C. obsoletus clade O1/C. montanus* *C. scoticus* *C. dewulfi*
50	07 Oct 2023	Nordhorn, Lower Saxony	cattle	SBV	31.8	Obsoletus Group	*C. obsoletus clade O1/C. montanus* *C. scoticus* *C. dewulfi*
50	07 Oct 2023	Nordhorn, Lower Saxony	cattle	SBV	34.5	Obsoletus Group	*C. obsoletus clade O1/C. montanus* *C. scoticus* *C. dewulfi*
50	07 Oct 2023	Nordhorn, Lower Saxony	cattle	SBV	34..1	Obsoletus Group	*C. obsoletus clade O1/C. montanus* *C. scoticus* *C. dewulfi*

Pool size, no. of biting midges	Collection date	Place of collection	Type of livestock	Virus	Cq value	Biting midge group/complex	Biting midge taxon
50	07 Oct 2023	Nordhorn, Lower Saxony	cattle	SBV	41.8	Obsoletus Group	*C. obsoletus clade O1/C. montanus*<br>*C. scoticus*<br>*C. dewulfi*<br>*C. chiopterus*
50	07 Oct 2023	Nordhorn, Lower Saxony	cattle	SBV	33.2	Obsoletus Group	*C. obsoletus clade O1/C. montanus*<br>*C. scoticus*<br>*C. dewulfi*<br>*C. chiopterus*
50	07 Oct 2023	Nordhorn, Lower Saxony	cattle	SBV	31.7	Obsoletus Group	*C. obsoletus clade O1/C. montanus*<br>*C. scoticus*<br>*C. dewulfi*
50	07 Oct 2023	Nordhorn, Lower Saxony	cattle	SBV	32.4	Obsoletus Group	*C. obsoletus clade O1/C. montanus*<br>*C. scoticus*<br>*C. dewulfi*
50	07 Oct 2023	Nordhorn, Lower Saxony	cattle	SBV	36.5	Obsoletus Group	*C. obsoletus clade O1/C. montanus*<br>*C. scoticus*<br>*C. dewulfi*<br>*C. chiopterus*
17	07 Oct 2023	Nordhorn, Lower Saxony	cattle	SBV	39.7	Obsoletus Group	*C. obsoletus clade O1/C. montanus*<br>*C. scoticus*<br>*C. dewulfi*
50	07 Oct 2023	Nordhorn, Lower Saxony	cattle	SBV	34.5	Obsoletus Group	*C. obsoletus clade O1/C. montanus*<br>*C. scoticus*<br>*C. dewulfi*<br>*C. chiopterus*
48	07 Oct 2023	Nordhorn, Lower Saxony	cattle	SBV	40.3	Obsoletus Group	*C. obsoletus clade O1/C. montanus*<br>*C. scoticus*<br>*C. dewulfi*<br>*C. chiopterus*
50	08 Oct 2023	Nordhorn, Lower Saxony	cattle	SBV	26.1	Obsoletus Group	*C. obsoletus clade O1/C. montanus*<br>*C. scoticus*<br>*C. dewulfi*<br>*C. chiopterus*
50	08 Oct 2023	Nordhorn, Lower Saxony	cattle	SBV	39.9	Obsoletus Group	*C. obsoletus clade O1/C. montanus*<br>*C. scoticus*
50	08 Oct 2023	Nordhorn, Lower Saxony	cattle	SBV	32.7	Obsoletus Group	*C. obsoletus clade O1/C. montanus*<br>*C. scoticus*<br>*C. dewulfi*<br>*C. chiopterus*
50	08 Oct 2023	Nordhorn, Lower Saxony	cattle	SBV	38.2	Obsoletus Group	*C. obsoletus clade O1/C. montanus*<br>*C. scoticus*<br>*C. chiopterus*
50	08 Oct 2023	Nordhorn, Lower Saxony	cattle	SBV	29.4	Obsoletus Group	*C. obsoletus clade O1/C. montanus*<br>*C. scoticus*<br>*C. dewulfi*<br>*C. chiopterus*
50	08 Oct 2023	Nordhorn, Lower Saxony	cattle	SBV	31.1	Obsoletus Group	*C. obsoletus clade O1/C. montanus*<br>*C. scoticus*<br>*C. dewulfi*
50	08 Oct 2023	Nordhorn, Lower Saxony	cattle	SBV	30.1	Obsoletus Group	*C. obsoletus clade O1/C. montanus*

Pool size, no. of biting midges	Collection date	Place of collection	Type of livestock	Virus	Cq value	Biting midge group/complex	Biting midge taxon
							C. scoticus<br>*C. chiopterus*
50	08 Oct 2023	Nordhorn, Lower Saxony	cattle	SBV	28..5	Obsoletus Group	*C. obsoletus clade O1/C. montanus*<br>*C. scoticus*<br>*C. dewulfi*
50	08 Oct 2023	Nordhorn, Lower Saxony	cattle	SBV	30.6	Obsoletus Group	*C. obsoletus clade O1/C. montanus*<br>*C. scoticus*<br>*C. dewulfi*<br>*C. chiopterus*
50	08 Oct 2023	Nordhorn, Lower Saxony	cattle	SBV	40.1	Obsoletus Group	*C. obsoletus clade O1/C. montanus*<br>*C. scoticus*<br>*C. dewulfi*
50	08 Oct 2023	Nordhorn, Lower Saxony	cattle	SBV	30.0	Obsoletus Group	*C. obsoletus clade O1/C. montanus*<br>*C. scoticus*
50	08 Oct 2023	Nordhorn, Lower Saxony	cattle	SBV	31.1	Obsoletus Group	*C. obsoletus clade O1/C. montanus*<br>*C. scoticus*<br>*C. dewulfi*
50	08 Oct 2023	Nordhorn, Lower Saxony	cattle	SBV	39.8	Obsoletus Group	*C. obsoletus clade O1/C. montanus*<br>*C. scoticus*<br>*C. dewulfi*
50	08 Oct 2023	Nordhorn, Lower Saxony	cattle	SBV	25.1	Obsoletus Group	*C. obsoletus clade O1/C. montanus*<br>*C. scoticus*
50	08 Oct 2023	Nordhorn, Lower Saxony	cattle	SBV	32.2	Obsoletus Group	*C. obsoletus clade O1/C. montanus*<br>*C. scoticus*<br>*C. dewulfi*<br>*C. chiopterus*
37	08 Oct 2023	Nordhorn, Lower Saxony	cattle	SBV	27.4	Obsoletus Group	*C. obsoletus clade O1/C. montanus*<br>*C. scoticus*
50	09 Oct 2023	Nordhorn, Lower Saxony	cattle	SBV	25.2	Obsoletus Group	*C. obsoletus clade O1/C. montanus*<br>*C. scoticus*<br>*C. chiopterus*
50	09 Oct 2023	Nordhorn, Lower Saxony	cattle	SBV	33.1	Obsoletus Group	*C. obsoletus clade O1/C. montanus*<br>*C. scoticus*<br>*C. dewulfi*<br>*C. chiopterus*
50	09 Oct 2023	Nordhorn, Lower Saxony	cattle	SBV	26.4	Obsoletus Group	*C. obsoletus clade O1/C. montanus*<br>*C. scoticus*<br>*C. dewulfi*<br>*C. chiopterus*
49	09 Oct 2023	Nordhorn, Lower Saxony	cattle	SBV	39.1	Obsoletus Group	*C. obsoletus clade O1/C. montanus*<br>*C. scoticus*<br>*C. dewulfi*<br>*C. chiopterus*
50	09 Oct 2023	Nordhorn, Lower Saxony	cattle	SBV	30.5	Obsoletus Group	*C. obsoletus clade O1/C. montanus*<br>*C. scoticus*<br>*C. chiopterus*
50	09 Oct 2023	Nordhorn, Lower Saxony	cattle	SBV	30.7	Obsoletus Group	*C. obsoletus clade O1/C. montanus*<br>*C. scoticus*<br>*C. chiopterus*

Pool size, no. of biting midges	Collection date	Place of collection	Type of livestock	Virus	Cq value	Biting midge group/complex	Biting midge taxon
50	09 Oct 2023	Nordhorn, Lower Saxony	cattle	SBV	31.0	Obsoletus Group	*C. obsoletus clade O1/C. montanus*<br>*C. scoticus*<br>*C. dewulfi*
50	09 Oct 2023	Nordhorn, Lower Saxony	cattle	SBV	40.6	Obsoletus Group	*C. obsoletus clade O1/C. montanus*<br>*C. scoticus*
50	09 Oct 2023	Nordhorn, Lower Saxony	cattle	SBV	26.7	Obsoletus Group	*C. obsoletus clade O1/C. montanus*<br>*C. scoticus*<br>*C. dewulfi*
50	09 Oct 2023	Nordhorn, Lower Saxony	cattle	SBV	31.1	Obsoletus Group	*C. obsoletus clade O1/C. montanus*<br>*C. scoticus*<br>*C. dewulfi*
50	09 Oct 2023	Nordhorn, Lower Saxony	cattle	SBV	39.4	Obsoletus Group	*C. obsoletus clade O1/C. montanus*<br>*C. scoticus*<br>*C. chiopterus*
50	09 Oct 2023	Nordhorn, Lower Saxony	cattle	SBV	29.8	Obsoletus Group	*C. obsoletus clade O1/C. montanus*<br>*C. scoticus*<br>*C. dewulfi*
50	09 Oct 2023	Nordhorn, Lower Saxony	cattle	SBV	38.9	Obsoletus Group	*C. obsoletus clade O1/C. montanus*<br>*C. scoticus*<br>*C. dewulfi*
50	09 Oct 2023	Nordhorn, Lower Saxony	cattle	SBV	28.5	Obsoletus Group	*C. obsoletus clade O1/C. montanus*<br>*C. scoticus*<br>*C. dewulfi*<br>*C. chiopterus*
50	09 Oct 2023	Nordhorn, Lower Saxony	cattle	SBV	27.3	Obsoletus Group	*C. obsoletus clade O1/C. montanus*<br>*C. scoticus*<br>*C. dewulfi*
50	09 Oct 2023	Nordhorn, Lower Saxony	cattle	SBV	40.8	Obsoletus Group	*C. obsoletus clade O1/C. montanus*<br>*C. scoticus*
50	09 Oct 2023	Nordhorn, Lower Saxony	cattle	SBV	33.8	Obsoletus Group	*C. obsoletus clade O1/C. montanus*<br>*C. scoticus*
50	09 Oct 2023	Nordhorn, Lower Saxony	cattle	SBV	33.8	Obsoletus Group	*C. obsoletus clade O1/C. montanus*<br>*C. scoticus*
31	09 Oct 2023	Nordhorn, Lower Saxony	cattle	SBV	34.1	Obsoletus Group	*C. obsoletus clade O1/C. montanus*<br>*C. scoticus*<br>*C. dewulfi*
50	10 Oct 2023	Nordhorn, Lower Saxony	cattle	SBV	34.0	Obsoletus Group	*C. obsoletus clade O1/C. montanus*<br>*C. scoticus*<br>*C. dewulfi*
50	10 Oct 2023	Nordhorn, Lower Saxony	cattle	SBV	36.7	Obsoletus Group	*C. obsoletus clade O1/C. montanus*<br>*C. scoticus*<br>*C. dewulfi*
50	10 Oct 2023	Nordhorn, Lower Saxony	cattle	SBV	37.3	Obsoletus Group	*C. obsoletus clade O1/C. montanus*<br>*C. scoticus*
50	10 Oct 2023	Nordhorn, Lower Saxony	cattle	SBV	37.3	Obsoletus Group	*C. obsoletus clade O1/C. montanus*<br>*C. scoticus*<br>*C. dewulfi*

Pool size, no. of biting midges	Collection date	Place of collection	Type of livestock	Virus	Cq value	Biting midge group/complex	Biting midge taxon
50	10 Oct 2023	Nordhorn, Lower Saxony	cattle	SBV	38.4	Obsoletus Group	*C. obsoletus clade O1/C. montanus*<br>*C. scoticus*<br>*C. dewulfi*
50	10 Oct 2023	Nordhorn, Lower Saxony	cattle	SBV	34.3	Obsoletus Group	*C. obsoletus clade O1/C. montanus*<br>*C. scoticus*<br>*C. dewulfi*
50	10 Oct 2023	Nordhorn, Lower Saxony	cattle	SBV	38.0	Obsoletus Group	*C. obsoletus clade O1/C. montanus*<br>*C. scoticus*<br>*C. dewulfi*
50	10 Oct 2023	Nordhorn, Lower Saxony	cattle	SBV	37.7	Obsoletus Group	*C. obsoletus clade O1/C. montanus*<br>*C. scoticus*<br>*C. chiopterus*
50	10 Oct 2023	Nordhorn, Lower Saxony	cattle	SBV	36.1	Obsoletus Group	*C. obsoletus clade O1/C. montanus*<br>*C. scoticus*<br>*C. dewulfi*
50	10 Oct 2023	Nordhorn, Lower Saxony	cattle	SBV	37.6	Obsoletus Group	*C. obsoletus clade O1/C. montanus*<br>*C. scoticus*
50	10 Oct 2023	Nordhorn, Lower Saxony	cattle	SBV	37.9	Obsoletus Group	*C. obsoletus clade O1/C. montanus*<br>*C. scoticus*<br>*C. dewulfi*
50	10 Oct 2023	Nordhorn, Lower Saxony	cattle	SBV	34.7	Obsoletus Group	*C. obsoletus clade O1/C. montanus*<br>*C. scoticus*<br>*C. dewulfi*
50	10 Oct 2023	Nordhorn, Lower Saxony	cattle	SBV	35.5	Obsoletus Group	*C. obsoletus clade O1/C. montanus*<br>*C. scoticus*<br>*C. dewulfi*
50	10 Oct 2023	Nordhorn, Lower Saxony	cattle	SBV	38.6	Obsoletus Group	*C. obsoletus clade O1/C. montanus*<br>*C. scoticus*<br>*C. dewulfi*
50	10 Oct 2023	Nordhorn, Lower Saxony	cattle	SBV	37.1	Obsoletus Group	*C. obsoletus clade O1/C. montanus*<br>*C. scoticus*<br>*C. dewulfi*
50	10 Oct 2023	Nordhorn, Lower Saxony	cattle	SBV	36.5	Obsoletus Group	*C. obsoletus clade O1/C. montanus*<br>*C. scoticus*
50	10 Oct 2023	Nordhorn, Lower Saxony	cattle	SBV	37.2	Obsoletus Group	*C. obsoletus clade O1/C. montanus*<br>*C. scoticus*<br>*C. dewulfi*
50	10 Oct 2023	Nordhorn, Lower Saxony	cattle	SBV	37.4	Obsoletus Group	*C. obsoletus clade O1/C. montanus*<br>*C. scoticus*<br>*C. dewulfi*<br>*C. chiopterus*
50	10 Oct 2023	Nordhorn, Lower Saxony	cattle	SBV	38.2	Obsoletus Group	*C. obsoletus clade O1/C. montanus*<br>*C. scoticus*<br>*C. chiopterus*
50	10 Oct 2023	Nordhorn, Lower Saxony	cattle	SBV	36.3	Obsoletus Group	*C. obsoletus clade O1/C. montanus*<br>*C. scoticus*<br>*C. dewulfi*
50	10 Oct 2023	Nordhorn, Lower Saxony	cattle	SBV	35.4	Obsoletus Group	*C. obsoletus clade O1/C. montanus*

Pool size, no. of biting midges	Collection date	Place of collection	Type of livestock	Virus	Cq value	Biting midge group/complex	Biting midge taxon
							C. scoticus
50	10 Oct 2023	Nordhorn, Lower Saxony	cattle	SBV	38.2	Obsoletus Group	*C. dewulfi*<br>*C. obsoletus clade O1/C. montanus*<br>*C. scoticus*
50	10 Oct 2023	Nordhorn, Lower Saxony	cattle	SBV	36.4	Obsoletus Group	*C. dewulfi*<br>*C. obsoletus clade O1/C. montanus*<br>*C. scoticus*
50	10 Oct 2023	Nordhorn, Lower Saxony	cattle	SBV	38.1	Obsoletus Group	*C. dewulfi*<br>*C. obsoletus clade O1/C. montanus*<br>*C. scoticus*
50	10 Oct 2023	Nordhorn, Lower Saxony	cattle	SBV	34.6	Obsoletus Group	*C. dewulfi*<br>*C. obsoletus clade O1/C. montanus*<br>*C. scoticus*
50	10 Oct 2023	Nordhorn, Lower Saxony	cattle	SBV	37.9	Obsoletus Group	*C. dewulfi*<br>*C. obsoletus clade O1/C. montanus*<br>*C. scoticus*
50	10 Oct 2023	Nordhorn, Lower Saxony	cattle	SBV	33.8	Obsoletus Group	*C. dewulfi*<br>*C. obsoletus clade O1/C. montanus*<br>*C. scoticus*
50	10 Oct 2023	Nordhorn, Lower Saxony	cattle	SBV	36.5	Obsoletus Group	*C. obsoletus clade O1/C. montanus*<br>*C. scoticus*
50	10 Oct 2023	Nordhorn, Lower Saxony	cattle	SBV	36.0	Obsoletus Group	*C. dewulfi*<br>*C. obsoletus clade O1/C. montanus*<br>*C. scoticus*
50	10 Oct 2023	Nordhorn, Lower Saxony	cattle	SBV	35.3	Obsoletus Group	*C. dewulfi*<br>*C. obsoletus clade O1/C. montanus*<br>*C. scoticus*
50	10 Oct 2023	Nordhorn, Lower Saxony	cattle	SBV	36.5	Obsoletus Group	*C. obsoletus clade O1/C. montanus*<br>*C. scoticus*
50	10 Oct 2023	Nordhorn, Lower Saxony	cattle	SBV	37.5	Obsoletus Group	*C. dewulfi*<br>*C. obsoletus clade O1/C. montanus*<br>*C. scoticus*
50	10 Oct 2023	Nordhorn, Lower Saxony	cattle	SBV	38.0	Obsoletus Group	*C. dewulfi*<br>*C. obsoletus clade O1/C. montanus*<br>*C. scoticus*
50	10 Oct 2023	Nordhorn, Lower Saxony	cattle	SBV	33.0	Obsoletus Group	*C. dewulfi*<br>*C. obsoletus clade O1/C. montanus*<br>*C. scoticus*
50	10 Oct 2023	Nordhorn, Lower Saxony	cattle	SBV	36.3	Obsoletus Group	*C. dewulfi*<br>*C. obsoletus clade O1/C. montanus*<br>*C. scoticus*
50	10 Oct 2023	Nordhorn, Lower Saxony	cattle	SBV	34.1	Obsoletus Group	*C. dewulfi*<br>*C. obsoletus clade O1/C. montanus*<br>*C. scoticus*
50	10 Oct 2023	Nordhorn, Lower Saxony	cattle	SBV	37.3	Obsoletus Group	*C. dewulfi*<br>*C. obsoletus clade O1/C. montanus*<br>*C. scoticus*
50	10 Oct 2023	Nordhorn, Lower Saxony	cattle	SBV	30.4	Obsoletus Group	*C. obsoletus clade O1/C. montanus*

Pool size, no. of biting midges	Collection date	Place of collection	Type of livestock	Virus	Cq value	Biting midge group/complex	Biting midge taxon
50	10 Oct 2023	Nordhorn, Lower Saxony	cattle	SBV	37.3	Obsoletus Group	*C. scoticus*<br>*C. dewulfi*<br>*C. obsoletus clade O1/C. montanus*
50	10 Oct 2023	Nordhorn, Lower Saxony	cattle	SBV	37.6	Obsoletus Group	*C. scoticus*<br>*C. dewulfi*<br>*C. obsoletus clade O1/C. montanus*
50	10 Oct 2023	Nordhorn, Lower Saxony	cattle	SBV	36.6	Obsoletus Group	*C. scoticus*<br>*C. dewulfi*<br>*C. obsoletus clade O1/C. montanus*
50	11 Oct 2023	Nordhorn, Lower Saxony	cattle	SBV	29.9	Obsoletus Group	*C. scoticus*<br>*C. obsoletus clade O1/C. montanus*
50	11 Oct 2023	Nordhorn, Lower Saxony	cattle	SBV	30.7	Obsoletus Group	*C. scoticus*<br>*C. chiopterus*<br>*C. obsoletus clade O1/C. montanus*
50	11 Oct 2023	Nordhorn, Lower Saxony	cattle	SBV	31.4	Obsoletus Group	*C. scoticus*<br>*C. dewulfi*<br>*C. obsoletus clade O1/C. montanus*
50	11 Oct 2023	Nordhorn, Lower Saxony	cattle	SBV	37.0	Obsoletus Group	*C. scoticus*<br>*C. dewulfi*<br>*C. obsoletus clade O1/C. montanus*
50	11 Oct 2023	Nordhorn, Lower Saxony	cattle	SBV	37.6	Obsoletus Group	*C. scoticus*<br>*C. dewulfi*<br>*C. obsoletus clade O1/C. montanus*
50	11 Oct 2023	Nordhorn, Lower Saxony	cattle	SBV	27.2	Obsoletus Group	*C. scoticus*<br>*C. dewulfi*<br>*C. chiopterus*<br>*C. obsoletus clade O1/C. montanus*
50	11 Oct 2023	Nordhorn, Lower Saxony	cattle	SBV	35.1	Obsoletus Group	*C. scoticus*<br>*C. dewulfi*<br>*C. obsoletus clade O1/C. montanus*
50	12 Oct 2023	Nordhorn, Lower Saxony	cattle	SBV	37.3	Obsoletus Group	*C. scoticus*<br>*C. dewulfi*<br>*C. obsoletus clade O1/C. montanus*
50	12 Oct 2023	Nordhorn, Lower Saxony	cattle	SBV	31.5	Obsoletus Group	*C. scoticus*<br>*C. dewulfi*<br>*C. obsoletus clade O1/C. montanus*
50	12 Oct 2023	Nordhorn, Lower Saxony	cattle	SBV	28.9	Obsoletus Group	*C. scoticus*<br>*C. dewulfi*<br>*C. chiopterus*<br>*C. obsoletus clade O1/C. montanus*
50	12 Oct 2023	Nordhorn, Lower Saxony	cattle	SBV	35.0	Obsoletus Group	*C. scoticus*<br>*C. dewulfi*<br>*C. obsoletus clade O1/C. montanus*
50	12 Oct 2023	Nordhorn, Lower Saxony	cattle	SBV	31.1	Obsoletus Group	*C. scoticus*<br>*C. dewulfi*<br>*C. chiopterus*<br>*C. obsoletus clade O1/C. montanus*
50	12 Oct 2023	Nordhorn, Lower Saxony	cattle	SBV	37.0	Obsoletus Group	*C. scoticus*<br>*C. obsoletus clade O1/C. montanus*

Pool size, no. of biting midges	Collection date	Place of collection	Type of livestock	Virus	Cq value	Biting midge group/complex	Biting midge taxon
							C. scoticus
50	03 Oct 2023	Kleve, North Rhine-Westphalia	cattle	SBV	33.4	Obsoletus Group	*C. obsoletus clade O1/C. montanus*<br>*C. scoticus*<br>*C. dewulfi*
50	03 Oct 2023	Kleve, North Rhine-Westphalia	cattle	SBV	28.5	Obsoletus Group	*C. obsoletus clade O1/C. montanus*<br>*C. scoticus*<br>*C. dewulfi*
50	04 Oct 2023	Kleve, North Rhine-Westphalia	cattle	SBV	32.2	Obsoletus Group	*C. obsoletus clade O1/C. montanus*<br>*C. scoticus*<br>*C. dewulfi*<br>*C. chiopterus*
50	08 Oct 2023	Kleve, North Rhine-Westphalia	cattle	SBV	30.7	Obsoletus Group	*C. obsoletus clade O1/C. montanus*<br>*C. scoticus*<br>*C. dewulfi*
50	09 Oct 2023	Kleve, North Rhine-Westphalia	cattle	SBV	30.2	Obsoletus Group	*C. obsoletus clade O1/C. montanus*<br>*C. scoticus*<br>*C. dewulfi*
50	09 Oct 2023	Kleve, North Rhine-Westphalia	cattle	SBV	30.0	Obsoletus Group	*C. obsoletus clade O1/C. montanus*<br>*C. scoticus*<br>*C. dewulfi*<br>*C. chiopterus*
50	10 Oct 2023	Kleve, North Rhine-Westphalia	cattle	SBV	29.1	Obsoletus Group	*C. obsoletus clade O1/C. montanus*<br>*C. scoticus*<br>*C. dewulfi*
50	10 Oct 2023	Kleve, North Rhine-Westphalia	cattle	SBV	34.1	Obsoletus Group	*C. obsoletus clade O1/C. montanus*<br>*C. scoticus*<br>*C. dewulfi*<br>*C. chiopterus*
50	10 Oct 2023	Kleve, North Rhine-Westphalia	cattle	SBV	31.9	Obsoletus Group	*C. obsoletus clade O1/C. montanus*<br>*C. scoticus*<br>*C. dewulfi*<br>*C. chiopterus*
50	10 Oct 2023	Kleve, North Rhine-Westphalia	cattle	SBV	28.3	Obsoletus Group	*C. obsoletus clade O1/C. montanus*<br>*C. scoticus*<br>*C. dewulfi*<br>*C. chiopterus*
50	02 Oct 2023	Kleve, North Rhine-Westphalia	cattle	SBV	27.2	Obsoletus Group	*C. obsoletus clade O1/C. montanus*<br>*C. scoticus*<br>*C. dewulfi*<br>*C. chiopterus*
50	02 Oct 2023	Kleve, North Rhine-Westphalia	cattle	SBV	39.6	Obsoletus Group	*C. obsoletus clade O1/C. montanus*<br>*C. scoticus*<br>*C. dewulfi*<br>*C. chiopterus*
50	02 Oct 2023	Kleve, North Rhine-Westphalia	cattle	SBV	26.2	Obsoletus Group	*C. obsoletus clade O1/C. montanus*<br>*C. scoticus*<br>*C. dewulfi*<br>*C. chiopterus*
50	13 Oct 2023	Kleve, North Rhine-Westphalia	cattle	SBV	28.6	Obsoletus Group	*C. obsoletus clade O1/C. montanus*<br>*C. scoticus*<br>*C. dewulfi*

Pool size, no. of biting midges	Collection date	Place of collection	Type of livestock	Virus	Cq value	Biting midge group/complex	Biting midge taxon
50	13 Oct 2023	Kleve, North Rhine-Westphalia	cattle	SBV	30.7	Obsoletus Group	*C. chiopterus*<br>*C. obsoletus clade O1/C. montanus*<br>*C. scoticus*
50	28 Sept 2023	Kleve, North Rhine-Westphalia	cattle	SBV	34.8	Obsoletus Group	*C. chiopterus*<br>*C. obsoletus clade O1/C. montanus*<br>*C. scoticus*
44	28 Sept 2023	Kleve, North Rhine-Westphalia	cattle	SBV	35.3	Obsoletus Group	*C. dewulfi*<br>*C. chiopterus*<br>*C. obsoletus clade O1/C. montanus*<br>*C. scoticus*
50	29 Sept 2023	Kleve, North Rhine-Westphalia	cattle	SBV	30.1	Obsoletus Group	*C. dewulfi*<br>*C. obsoletus clade O1/C. montanus*<br>*C. scoticus*
50	29 Sept 2023	Kleve, North Rhine-Westphalia	cattle	SBV	27.4	Obsoletus Group	*C. dewulfi*<br>*C. chiopterus*<br>*C. obsoletus clade O1/C. montanus*<br>*C. scoticus*
50	24 Oct 2023	Kleve, North Rhine-Westphalia	cattle	SBV	33.4	Obsoletus Group	*C. dewulfi*<br>*C. obsoletus clade O1/C. montanus*<br>*C. scoticus*
50	13 Oct 2023	Gronau, North Rhine-Westphalia	cattle	SBV	35.5	Obsoletus Group	*C. chiopterus*<br>*C. obsoletus clade O1/C. montanus*<br>*C. scoticus*
50	13 Oct 2023	Gronau, North Rhine-Westphalia	cattle	SBV	26.9	Obsoletus Group	*C. dewulfi*<br>*C. obsoletus clade O1/C. montanus*<br>*C. scoticus*
50	13 Oct 2023	Gronau, North Rhine-Westphalia	cattle	SBV	28.9	Obsoletus Group	*C. dewulfi*<br>*C. chiopterus*<br>*C. obsoletus clade O1/C. montanus*<br>*C. scoticus*
50	13 Oct 2023	Gronau, North Rhine-Westphalia	cattle	SBV	35.1	Obsoletus Group	*C. dewulfi*<br>*C. chiopterus*<br>*C. obsoletus clade O1/C. montanus*<br>*C. scoticus*
50	13 Oct 2023	Gronau, North Rhine-Westphalia	cattle	SBV	31.2	Obsoletus Group	*C. dewulfi*<br>*C. chiopterus*<br>*C. obsoletus clade O1/C. montanus*<br>*C. scoticus*
50	13 Oct 2023	Gronau, North Rhine-Westphalia	cattle	SBV	29.4	Obsoletus Group	*C. dewulfi*<br>*C. chiopterus*<br>*C. obsoletus clade O1/C. montanus*<br>*C. scoticus*
50	13 Oct 2023	Gronau, North Rhine-Westphalia	cattle	SBV	34.5	Obsoletus Group	*C. dewulfi*<br>*C. obsoletus clade O1/C. montanus*<br>*C. scoticus*
50	13 Oct 2023	Gronau, North Rhine-Westphalia	cattle	SBV	34.7	Obsoletus Group	*C. dewulfi*<br>*C. obsoletus clade O1/C. montanus*<br>*C. scoticus*<br>*C. dewulfi*<br>*C. chiopterus*

Pool size, no. of biting midges	Collection date	Place of collection	Type of livestock	Virus	Cq value	Biting midge group/complex	Biting midge taxon
50	13 Oct 2023	Gronau, North Rhine-Westphalia	cattle	SBV	29.2	Obsoletus Group	*C. obsoletus clade O1/C. montanus* *C. scoticus* *C. dewulfi* *C. chiopterus*
50	13 Oct 2023	Gronau, North Rhine-Westphalia	cattle	SBV	32.7	Obsoletus Group	*C. obsoletus clade O1/C. montanus* *C. scoticus* *C. dewulfi*
50	13 Oct 2023	Gronau, North Rhine-Westphalia	cattle	SBV	34.7	Obsoletus Group	*C. obsoletus clade O1/C. montanus* *C. scoticus* *C. dewulfi* *C. chiopterus*
50	13 Oct 2023	Gronau, North Rhine-Westphalia	cattle	SBV	34.3	Obsoletus Group	*C. obsoletus clade O1/C. montanus* *C. scoticus* *C. dewulfi* *C. chiopterus*
50	13 Oct 2023	Gronau, North Rhine-Westphalia	cattle	SBV	26.1	Obsoletus Group	*C. obsoletus clade O1/C. montanus* *C. scoticus* *C. dewulfi*
50	13 Oct 2023	Gronau, North Rhine-Westphalia	cattle	SBV	36.0	Obsoletus Group	*C. obsoletus clade O1/C. montanus* *C. scoticus* *C. dewulfi* *C. chiopterus*
50	13 Oct 2023	Gronau, North Rhine-Westphalia	cattle	SBV	29.9	Obsoletus Group	*C. obsoletus clade O1/C. montanus* *C. scoticus* *C. dewulfi* *C. chiopterus*
50	13 Oct 2023	Gronau, North Rhine-Westphalia	cattle	SBV	35.2	Obsoletus Group	*C. obsoletus clade O1/C. montanus* *C. scoticus* *C. dewulfi*
50	13 Oct 2023	Gronau, North Rhine-Westphalia	cattle	SBV	35.0	Obsoletus Group	*C. obsoletus clade O1/C. montanus* *C. scoticus* *C. dewulfi*
50	13 Oct 2023	Gronau, North Rhine-Westphalia	cattle	SBV	34.6	Obsoletus Group	*C. obsoletus clade O1/C. montanus* *C. scoticus* *C. dewulfi* *C. chiopterus*
50	13 Oct 2023	Gronau, North Rhine-Westphalia	cattle	SBV	35.2	Obsoletus Group	*C. obsoletus clade O1/C. montanus* *C. scoticus* *C. dewulfi*
50	13 Oct 2023	Gronau, North Rhine-Westphalia	cattle	SBV	34.4	Obsoletus Group	*C. obsoletus clade O1/C. montanus* *C. scoticus* *C. dewulfi* *C. chiopterus*
50	13 Oct 2023	Gronau, North Rhine-Westphalia	cattle	SBV	35.2	Obsoletus Group	*C. obsoletus clade O1/C. montanus* *C. scoticus* *C. dewulfi*
8	13 Oct 2023	Gronau, North Rhine-Westphalia	cattle	SBV	37.2	Obsoletus Group	*C. obsoletus clade O1/C. montanus* *C. scoticus*
50	13 Oct 2023	Gronau, North Rhine-Westphalia	cattle	SBV	38.8	Obsoletus Group	*C. obsoletus clade O1/C. montanus* *C. scoticus*

Pool size, no. of biting midges	Collection date	Place of collection	Type of livestock	Virus	Cq value	Biting midge group/complex	Biting midge taxon
							C. dewulfi
50	13 Oct 2023	Gronau, North Rhine-Westphalia	cattle	SBV	30.2	Obsoletus Group	C. obsoletus clade O1/C. montanus<br>C. scoticus<br>C. dewulfi
50	13 Oct 2023	Gronau, North Rhine-Westphalia	cattle	SBV	36.2	Obsoletus Group	C. obsoletus clade O1/C. montanus<br>C. scoticus<br>C. dewulfi
36	13 Oct 2023	Gronau, North Rhine-Westphalia	cattle	SBV	31.5	Obsoletus Group	C. obsoletus clade O1/C. montanus<br>C. scoticus<br>C. dewulfi
23	13 Oct 2023	Gronau 2, North Rhine-Westphalia	cattle	SBV	39.1	Pulicaris Complex	C. punctatus<br>C. pulicaris
50	12 Oct 2023	Gronau 2, North Rhine-Westphalia	cattle	SBV	29.7	Obsoletus Group	C. obsoletus clade O1/C. montanus<br>C. scoticus<br>C. dewulfi<br>C. chiopterus
50	12 Oct 2023	Gronau 2, North Rhine-Westphalia	cattle	SBV	29.4	Obsoletus Group	C. obsoletus clade O1/C. montanus<br>C. scoticus<br>C. dewulfi<br>C. chiopterus
35	12 Oct 2023	Gronau 2, North Rhine-Westphalia	cattle	SBV	25.2	Obsoletus Group	C. obsoletus clade O1/C. montanus<br>C. scoticus<br>C. chiopterus
50	13 Oct 2023	Gronau 2, North Rhine-Westphalia	cattle	SBV	28.5	Obsoletus Group	C. obsoletus clade O1/C. montanus<br>C. scoticus<br>C. dewulfi<br>C. chiopterus
50	13 Oct 2023	Gronau 2, North Rhine-Westphalia	cattle	SBV	34.4	Obsoletus Group	C. obsoletus clade O1/C. montanus<br>C. scoticus<br>C. dewulfi<br>C. chiopterus
48	13 Oct 2023	Gronau 2, North Rhine-Westphalia	cattle	SBV	30.1	Obsoletus Group	C. obsoletus clade O1/C. montanus<br>C. scoticus<br>C. dewulfi<br>C. chiopterus
11	13 Oct 2023	Gronau 2, North Rhine-Westphalia	cattle	SBV	28.3	Pulicaris Complex	C. punctatus<br>C. pulicaris
50	14 Oct 2023	Gronau 2, North Rhine-Westphalia	cattle	SBV	28.0	Obsoletus Group	C. obsoletus clade O1/C. montanus<br>C. scoticus<br>C. chiopterus
10	16 Oct 2023	Rees, North Rhine-Westphalia	cattle	SBV	25.7	Obsoletus Group	C. obsoletus clade O1/C. montanus<br>C. scoticus<br>C. dewulfi
20	13 Oct 2023	Kalkar, North Rhine-Westphalia	cattle	SBV	39.6	Obsoletus Group	C. obsoletus clade O1/C. montanus<br>C. scoticus
50	14 Oct 2023	Kalkar, North Rhine-Westphalia	cattle	SBV	24.1	Obsoletus Group	C. obsoletus clade O1/C. montanus<br>C. scoticus<br>C. dewulfi
42	29 Oct 2023	Hünxe, North Rhine-Westphalia	sheep, goat	SBV	29.3	Obsoletus Group	C. obsoletus clade O1/C. montanus<br>C. scoticus

Pool size, no. of biting midges	Collection date	Place of collection	Type of livestock	Virus	Cq value	Biting midge group/complex	Biting midge taxon
50	09 Oct 2023	Hünxe, North Rhine-Westphalia	sheep, goat	SBV	23.7	Obsoletus Group	*C. obsoletus clade O1/C. montanus C. scoticus C. dewulfi*
50	09 Oct 2023	Hünxe, North Rhine-Westphalia	sheep, goat	SBV	39.2	Obsoletus Group	*C. obsoletus clade O1/C. montanus C. scoticus*
50	11 Oct 2023	Hünxe, North Rhine-Westphalia	sheep, goat	SBV	25.9	Obsoletus Group	*C. obsoletus clade O1/C. montanus C. scoticus C. dewulfi*
50	03 Oct 2023	Neukirchen, North Rhine-Westphalia	goat	SBV	27.6	Obsoletus Group	*C. obsoletus clade O1/C. montanus C. scoticus*
50	2023-09-28	Selfkant, North Rhine-Westphalia	cattle	SBV	33.8	Obsoletus Group	*C. obsoletus clade O1/C. montanus C. scoticus C. dewulfi C. chiopterus*
50	28 Sept 2023	Selfkant, North Rhine-Westphalia	cattle	SBV	34.8	Obsoletus Group	*C. obsoletus clade O1/C. montanus C. scoticus C. dewulfi C. chiopterus*
50	28 Sept 2023	Selfkant, North Rhine-Westphalia	cattle	SBV	34.2	Obsoletus Group	*C. obsoletus clade O1/C. montanus C. scoticus C. dewulfi*
50	28 Sept 2023	Selfkant, North Rhine-Westphalia	cattle	SBV	34.5	Obsoletus Group	*C. obsoletus clade O1/C. montanus C. scoticus C. dewulfi*
50	28 Sept 2023	Selfkant, North Rhine-Westphalia	cattle	SBV	30.3	Obsoletus Group	*C. obsoletus clade O1/C. montanus C. scoticus C. dewulfi*
50	28 Sept 2023	Selfkant, North Rhine-Westphalia	cattle	SBV	35.3	Obsoletus Group	*C. obsoletus clade O1/C. montanus C. scoticus C. dewulfi*
50	28 Sept 2023	Selfkant, North Rhine-Westphalia	cattle	SBV	33.2	Obsoletus Group	*C. obsoletus clade O1/C. montanus C. scoticus C. dewulfi*
50	28 Sept 2023	Selfkant, North Rhine-Westphalia	cattle	SBV	34.0	Obsoletus Group	*C. obsoletus clade O1/C. montanus C. scoticus C. dewulfi*
50	28 Sept 2023	Selfkant, North Rhine-Westphalia	cattle	SBV	34.0	Obsoletus Group	*C. obsoletus clade O1/C. montanus C. scoticus C. dewulfi*
50	28 Sept 2023	Selfkant, North Rhine-Westphalia	cattle	SBV	33.7	Obsoletus Group	*C. obsoletus clade O1/C. montanus C. scoticus C. dewulfi*
50	28 Sept 2023	Selfkant, North Rhine-Westphalia	cattle	SBV	33.4	Obsoletus Group	*C. obsoletus clade O1/C. montanus C. scoticus C. dewulfi*
50	28 Sept 2023	Selfkant, North Rhine-Westphalia	cattle	SBV	33.2	Obsoletus Group	*C. obsoletus clade O1/C. montanus C. scoticus C. dewulfi*

Pool size, no. of biting midges	Collection date	Place of collection	Type of livestock	Virus	Cq value	Biting midge group/complex	Biting midge taxon
50	28 Sept 2023	Selfkant, North Rhine-Westphalia	cattle	SBV	33.2	Obsoletus Group	*C. obsoletus clade O1/C. montanus C. scoticus C. dewulfi*
50	28 Sept 2023	Selfkant, North Rhine-Westphalia	cattle	SBV	33.9	Obsoletus Group	*C. obsoletus clade O1/C. montanus C. scoticus C. dewulfi*
50	28 Sept 2023	Selfkant, North Rhine-Westphalia	cattle	SBV	33.5	Obsoletus Group	*C. obsoletus clade O1/C. montanus C. scoticus C. dewulfi*
50	28 Sept 2023	Selfkant, North Rhine-Westphalia	cattle	SBV	27.1	Obsoletus Group	*C. obsoletus clade O1/C. montanus C. scoticus C. dewulfi*
50	28 Sept 2023	Selfkant, North Rhine-Westphalia	cattle	SBV	34.6	Obsoletus Group	*C. obsoletus clade O1/C. montanus C. scoticus C. dewulfi*
50	28 Sept 2023	Selfkant, North Rhine-Westphalia	cattle	SBV	33.6	Obsoletus Group	*C. obsoletus clade O1/C. montanus C. scoticus C. dewulfi*
50	28 Sept 2023	Selfkant, North Rhine-Westphalia	cattle	SBV	34.1	Obsoletus Group	*C. obsoletus clade O1/C. montanus C. scoticus C. dewulfi*
50	30 Sept 2023	Selfkant, North Rhine-Westphalia	cattle	SBV	38.5	Obsoletus Group	*C. obsoletus clade O1/C. montanus C. scoticus C. dewulfi*
50	30 Sept 2023	Selfkant, North Rhine-Westphalia	cattle	SBV	38.2	Obsoletus Group	*C. obsoletus clade O1/C. montanus C. scoticus C. dewulfi*
50	30 Sept 2023	Selfkant, North Rhine-Westphalia	cattle	SBV	24.6	Obsoletus Group	*C. obsoletus clade O1/C. montanus C. scoticus C. dewulfi*
50	30 Sept 2023	Selfkant, North Rhine-Westphalia	cattle	SBV	32.7	Obsoletus Group	*C. obsoletus clade O1/C. montanus C. scoticus C. dewulfi*
50	30 Sept 2023	Selfkant, North Rhine-Westphalia	cattle	SBV	39.2	Obsoletus Group	*C. obsoletus clade O1/C. montanus C. scoticus C. dewulfi*
50	30 Sept 2023	Selfkant, North Rhine-Westphalia	cattle	SBV	29.7	Obsoletus Group	*C. obsoletus clade O1/C. montanus C. scoticus C. dewulfi*
50	30 Sept 2023	Selfkant, North Rhine-Westphalia	cattle	SBV	38.5	Obsoletus Group	*C. obsoletus clade O1/C. montanus C. scoticus C. dewulfi C. chiopterus*
50	30 Sept 2023	Selfkant, North Rhine-Westphalia	cattle	SBV	37.8	Obsoletus Group	*C. obsoletus clade O1/C. montanus C. scoticus C. dewulfi*
50	30 Sept 2023	Selfkant, North Rhine-Westphalia	cattle	SBV	38.1	Obsoletus Group	*C. obsoletus clade O1/C. montanus C. scoticus C. dewulfi*

Pool size, no. of biting midges	Collection date	Place of collection	Type of livestock	Virus	Cq value	Biting midge group/complex	Biting midge taxon
50	30 Sept 2023	Selfkant, North Rhine-Westphalia	cattle	SBV	38.4	Obsoletus Group	*C. obsoletus clade O1/C. montanus*<br>*C. scoticus*<br>*C. dewulfi*
50	30 Sept 2023	Selfkant, North Rhine-Westphalia	cattle	SBV	31.1	Obsoletus Group	*C. obsoletus clade O1/C. montanus*<br>*C. scoticus*<br>*C. dewulfi*
50	30 Sept 2023	Selfkant, North Rhine-Westphalia	cattle	SBV	38.8	Obsoletus Group	*C. obsoletus clade O1/C. montanus*<br>*C. scoticus*<br>*C. dewulfi*
50	30 Sept 2023	Selfkant, North Rhine-Westphalia	cattle	SBV	36.9	Obsoletus Group	*C. obsoletus clade O1/C. montanus*<br>*C. scoticus*<br>*C. dewulfi*<br>*C. chiopterus*
50	30 Sept 2023	Selfkant, North Rhine-Westphalia	cattle	SBV	37.9	Obsoletus Group	*C. obsoletus clade O1/C. montanus*<br>*C. scoticus*<br>*C. dewulfi*
50	30 Sept 2023	Selfkant, North Rhine-Westphalia	cattle	SBV	39.1	Obsoletus Group	*C. obsoletus clade O1/C. montanus*<br>*C. scoticus*<br>*C. dewulfi*
50	30 Sept 2023	Selfkant, North Rhine-Westphalia	cattle	SBV	38.0	Obsoletus Group	*C. obsoletus clade O1/C. montanus*<br>*C. scoticus*<br>*C. dewulfi*
50	30 Sept 2023	Selfkant, North Rhine-Westphalia	cattle	SBV	37.6	Obsoletus Group	*C. obsoletus clade O1/C. montanus*<br>*C. scoticus*<br>*C. dewulfi*
50	30 Sept 2023	Selfkant, North Rhine-Westphalia	cattle	SBV	37.5	Obsoletus Group	*C. obsoletus clade O1/C. montanus*<br>*C. scoticus*<br>*C. dewulfi*
50	30 Sept 2023	Selfkant, North Rhine-Westphalia	cattle	SBV	38.7	Obsoletus Group	*C. obsoletus clade O1/C. montanus*<br>*C. scoticus*<br>*C. dewulfi*
50	30 Sept 2023	Selfkant, North Rhine-Westphalia	cattle	SBV	39.3	Obsoletus Group	*C. obsoletus clade O1/C. montanus*<br>*C. scoticus*<br>*C. dewulfi*
50	30 Sept 2023	Selfkant, North Rhine-Westphalia	cattle	SBV	39.0	Obsoletus Group	*C. obsoletus clade O1/C. montanus*<br>*C. scoticus*<br>*C. dewulfi*
50	30 Sept 2023	Selfkant, North Rhine-Westphalia	cattle	SBV	38.2	Obsoletus Group	*C. obsoletus clade O1/C. montanus*<br>*C. scoticus*<br>*C. dewulfi*
50	30 Sept 2023	Selfkant, North Rhine-Westphalia	cattle	SBV	38.9	Obsoletus Group	*C. obsoletus clade O1/C. montanus*<br>*C. scoticus*<br>*C. dewulfi*
50	30 Sept 2023	Selfkant, North Rhine-Westphalia	cattle	SBV	39.8	Obsoletus Group	*C. obsoletus clade O1/C. montanus*<br>*C. scoticus*<br>*C. dewulfi*
50	30 Sept 2023	Selfkant, North Rhine-Westphalia	cattle	SBV	29.0	Obsoletus Group	*C. obsoletus clade O1/C. montanus*<br>*C. scoticus*<br>*C. dewulfi*

Pool size, no. of biting midges	Collection date	Place of collection	Type of livestock	Virus	Cq value	Biting midge group/complex	Biting midge taxon
50	30 Sept 2023	Selfkant, North Rhine-Westphalia	cattle	SBV	23.9	Obsoletus Group	*C. obsoletus clade O1/C. montanus* *C. scoticus* *C. dewulfi*
50	03 Oct 2023	Selfkant, North Rhine-Westphalia	cattle	SBV	36.6	Obsoletus Group	*C. obsoletus clade O1/C. montanus* *C. scoticus*
50	03 Oct 2023	Selfkant, North Rhine-Westphalia	cattle	SBV	31.2	Obsoletus Group	*C. obsoletus clade O1/C. montanus* *C. scoticus* *C. dewulfi*
50	03 Oct 2023	Selfkant, North Rhine-Westphalia	cattle	SBV	35.9	Obsoletus Group	*C. obsoletus clade O1/C. montanus* *C. scoticus* *C. dewulfi*
50	03 Oct 2023	Selfkant, North Rhine-Westphalia	cattle	SBV	38.2	Obsoletus Group	*C. obsoletus clade O1/C. montanus* *C. scoticus* *C. dewulfi*
50	03 Oct 2023	Selfkant, North Rhine-Westphalia	cattle	SBV	35.1	Obsoletus Group	*C. obsoletus clade O1/C. montanus* *C. scoticus* *C. dewulfi*
50	03 Oct 2023	Selfkant, North Rhine-Westphalia	cattle	SBV	34.3	Obsoletus Group	*C. obsoletus clade O1/C. montanus* *C. scoticus* *C. dewulfi*
50	03 Oct 2023	Selfkant, North Rhine-Westphalia	cattle	SBV	28.1	Obsoletus Group	*C. obsoletus clade O1/C. montanus* *C. scoticus* *C. dewulfi*
50	03 Oct 2023	Selfkant, North Rhine-Westphalia	cattle	SBV	37.1	Obsoletus Group	*C. obsoletus clade O1/C. montanus* *C. scoticus* *C. dewulfi*
50	03 Oct 2023	Selfkant, North Rhine-Westphalia	cattle	SBV	28.3	Obsoletus Group	*C. obsoletus clade O1/C. montanus* *C. scoticus* *C. dewulfi*
50	03 Oct 2023	Selfkant, North Rhine-Westphalia	cattle	SBV	36.9	Obsoletus Group	*C. obsoletus clade O1/C. montanus* *C. scoticus* *C. dewulfi*
50	03 Oct 2023	Selfkant, North Rhine-Westphalia	cattle	SBV	37.9	Obsoletus Group	*C. obsoletus clade O1/C. montanus* *C. scoticus* *C. dewulfi*
50	03 Oct 2023	Selfkant, North Rhine-Westphalia	cattle	SBV	31.5	Obsoletus Group	*C. obsoletus clade O1/C. montanus* *C. scoticus* *C. dewulfi*
50	03 Oct 2023	Selfkant, North Rhine-Westphalia	cattle	SBV	37.1	Obsoletus Group	*C. obsoletus clade O1/C. montanus* *C. scoticus* *C. dewulfi*
50	03 Oct 2023	Selfkant, North Rhine-Westphalia	cattle	SBV	38.2	Obsoletus Group	*C. obsoletus clade O1/C. montanus* *C. scoticus* *C. dewulfi*
50	03 Oct 2023	Selfkant, North Rhine-Westphalia	cattle	SBV	35.7	Obsoletus Group	*C. obsoletus clade O1/C. montanus* *C. scoticus* *C. dewulfi*
50	03 Oct 2023	Selfkant, North Rhine-Westphalia	cattle	SBV	34.4	Obsoletus Group	*C. obsoletus clade O1/C. montanus*

Pool size, no. of biting midges	Collection date	Place of collection	Type of livestock	Virus	Cq value	Biting midge group/complex	Biting midge taxon
							C. scoticus
50	03 Oct 2023	Selfkant, North Rhine-Westphalia	cattle	SBV	37.9	Obsoletus Group	*C. obsoletus clade O1/C. montanus C. scoticus C. dewulfi*
50	03 Oct 2023	Selfkant, North Rhine-Westphalia	cattle	SBV	25.5	Obsoletus Group	*C. obsoletus clade O1/C. montanus C. scoticus C. dewulfi*
50	03 Oct 2023	Selfkant, North Rhine-Westphalia	cattle	SBV	38.9	Obsoletus Group	*C. obsoletus clade O1/C. montanus C. scoticus C. dewulfi*
50	03 Oct 2023	Selfkant, North Rhine-Westphalia	cattle	SBV	24.2	Obsoletus Group	*C. obsoletus clade O1/C. montanus C. scoticus C. dewulfi*
50	03 Oct 2023	Selfkant, North Rhine-Westphalia	cattle	SBV	35.6	Obsoletus Group	*C. obsoletus clade O1/C. montanus C. scoticus C. dewulfi*
50	03 Oct 2023	Selfkant, North Rhine-Westphalia	cattle	SBV	37.3	Obsoletus Group	*C. obsoletus clade O1/C. montanus C. scoticus C. dewulfi*
50	03 Oct 2023	Selfkant, North Rhine-Westphalia	cattle	SBV	28.4	Obsoletus Group	*C. obsoletus clade O1/C. montanus C. scoticus C. dewulfi*
50	03 Oct 2023	Selfkant, North Rhine-Westphalia	cattle	SBV	38.8	Obsoletus Group	*C. obsoletus clade O1/C. montanus C. scoticus C. dewulfi*
50	03 Oct 2023	Selfkant, North Rhine-Westphalia	cattle	SBV	36.5	Obsoletus Group	*C. obsoletus clade O1/C. montanus C. scoticus C. dewulfi*
50	03 Oct 2023	Selfkant, North Rhine-Westphalia	cattle	SBV	36.0	Obsoletus Group	*C. obsoletus clade O1/C. montanus C. scoticus C. dewulfi*
50	03 Oct 2023	Selfkant, North Rhine-Westphalia	cattle	SBV	36.3	Obsoletus Group	*C. obsoletus clade O1/C. montanus C. scoticus C. dewulfi*
50	03 Oct 2023	Selfkant, North Rhine-Westphalia	cattle	SBV	34.9	Obsoletus Group	*C. obsoletus clade O1/C. montanus C. scoticus C. dewulfi*
50	03 Oct 2023	Selfkant, North Rhine-Westphalia	cattle	SBV	39.6	Obsoletus Group	*C. obsoletus clade O1/C. montanus C. scoticus C. dewulfi*
50	03 Oct 2023	Selfkant, North Rhine-Westphalia	cattle	SBV	37.7	Obsoletus Group	*C. obsoletus clade O1/C. montanus C. scoticus C. dewulfi*
50	03 Oct 2023	Selfkant, North Rhine-Westphalia	cattle	SBV	32.4	Obsoletus Group	*C. obsoletus clade O1/C. montanus C. scoticus C. dewulfi*
50	03 Oct 2023	Selfkant, North Rhine-Westphalia	cattle	SBV	24.0	Obsoletus Group	*C. obsoletus clade O1/C. montanus C. scoticus C. dewulfi*

Pool size, no. of biting midges	Collection date	Place of collection	Type of livestock	Virus	Cq value	Biting midge group/complex	Biting midge taxon
50	03 Oct 2023	Selfkant, North Rhine-Westphalia	cattle	SBV	38.8	Obsoletus Group	*C. obsoletus clade O1/C. montanus* *C. scoticus* *C. dewulfi*
50	03 Oct 2023	Selfkant, North Rhine-Westphalia	cattle	SBV	36.3	Obsoletus Group	*C. obsoletus clade O1/C. montanus* *C. scoticus* *C. dewulfi*
50	03 Oct 2023	Selfkant, North Rhine-Westphalia	cattle	SBV	36.4	Obsoletus Group	*C. obsoletus clade O1/C. montanus* *C. scoticus* *C. dewulfi*
50	03 Oct 2023	Selfkant, North Rhine-Westphalia	cattle	SBV	37.9	Obsoletus Group	*C. obsoletus clade O1/C. montanus* *C. scoticus* *C. dewulfi*
50	03 Oct 2023	Selfkant, North Rhine-Westphalia	cattle	SBV	36.6	Obsoletus Group	*C. obsoletus clade O1/C. montanus* *C. scoticus* *C. dewulfi*
50	03 Oct 2023	Selfkant, North Rhine-Westphalia	cattle	SBV	37.6	Obsoletus Group	*C. obsoletus clade O1/C. montanus* *C. scoticus* *C. dewulfi*
50	03 Oct 2023	Selfkant, North Rhine-Westphalia	cattle	SBV	38.9	Obsoletus Group	*C. obsoletus clade O1/C. montanus* *C. scoticus* *C. dewulfi*
50	03 Oct 2023	Selfkant, North Rhine-Westphalia	cattle	SBV	27.5	Obsoletus Group	*C. obsoletus clade O1/C. montanus* *C. scoticus* *C. dewulfi*
50	03 Oct 2023	Selfkant, North Rhine-Westphalia	cattle	SBV	25.7	Obsoletus Group	*C. obsoletus clade O1/C. montanus* *C. scoticus* *C. dewulfi*
50	03 Oct 2023	Nordhorn, Lower Saxony	cattle	SBV	28.5	Obsoletus Group	*C. obsoletus clade O1/C. montanus* *C. scoticus* *C. chiopterus*
50	03 Oct 2023	Nordhorn, Lower Saxony	cattle	SBV	39.0	Obsoletus Group	*C. obsoletus clade O1/C. montanus* *C. scoticus* *C. dewulfi*
50	03 Oct 2023	Nordhorn, Lower Saxony	cattle	SBV	39.6	Obsoletus Group	*C. obsoletus clade O1/C. montanus* *C. scoticus* *C. chiopterus*
50	03 Oct 2023	Nordhorn, Lower Saxony	cattle	SBV	35.4	Obsoletus Group	*C. obsoletus clade O1/C. montanus* *C. scoticus*
50	03 Oct 2023	Nordhorn, Lower Saxony	cattle	SBV	36.4	Obsoletus Group	*C. obsoletus clade O1/C. montanus* *C. scoticus* *C. dewulfi*
50	03 Oct 2023	Nordhorn, Lower Saxony	cattle	SBV	39.9	Obsoletus Group	*C. obsoletus clade O1/C. montanus* *C. scoticus* *C. dewulfi*
50	03 Oct 2023	Nordhorn, Lower Saxony	cattle	SBV	30.8	Obsoletus Group	*C. obsoletus clade O1/C. montanus* *C. scoticus* *C. chiopterus*
50	03 Oct 2023	Nordhorn, Lower Saxony	cattle	SBV	34.4	Obsoletus Group	*C. obsoletus clade O1/C. montanus*

Pool size, no. of biting midges	Collection date	Place of collection	Type of livestock	Virus	Cq value	Biting midge group/complex	Biting midge taxon
							C. scoticus
50	03 Oct 2023	Nordhorn, Lower Saxony	cattle	SBV	31.2	Obsoletus Group	*C. obsoletus clade O1/C. montanus*<br>*C. scoticus*
50	03 Oct 2023	Nordhorn, Lower Saxony	cattle	SBV	31.6	Obsoletus Group	*C. obsoletus clade O1/C. montanus*<br>*C. scoticus*<br>*C. chiopterus*
9	01 Oct 2023	Neukirchen, North Rhine-Westphalia	goat	SBV	39.9	Obsoletus Group	*C. obsoletus clade O1/C. montanus*<br>*C. scoticus*<br>*C. dewulfi*
47	09 Nov 2023	Neukirchen, North Rhine-Westphalia	goat	SBV	25.4	Obsoletus Group	*C. obsoletus clade O1/C. montanus*<br>*C. scoticus*<br>*C. dewulfi*
50	29 Sept 2023	Selfkant, North Rhine-Westphalia	cattle	SBV	38.1	Obsoletus Group	*C. obsoletus clade O1/C. montanus*<br>*C. scoticus*<br>*C. dewulfi*<br>*C. chiopterus*
50	29 Sept 2023	Selfkant, North Rhine-Westphalia	cattle	SBV	38.3	Obsoletus Group	*C. obsoletus clade O1/C. montanus*<br>*C. scoticus*<br>*C. dewulfi*
50	29 Sept 2023	Selfkant, North Rhine-Westphalia	cattle	SBV	38.0	Obsoletus Group	*C. obsoletus clade O1/C. montanus*<br>*C. scoticus*<br>*C. dewulfi*
50	28 Sept 2023	Selfkant, North Rhine-Westphalia	cattle	SBV	34.5	Pulicaris Complex	*C. pulicaris*<br>*C. lupicaris clade L1*<br>*C. newsteadi clade N1*
50	28 Sept 2023	Selfkant, North Rhine-Westphalia	cattle	SBV	35.7	Pulicaris Complex	*C. punctatus*<br>*C. pulicaris*<br>*C. lupicaris clade L1*<br>*C. newsteadi clade N1*
50	28 Sept 2023	Selfkant, North Rhine-Westphalia	cattle	SBV	35.4	Pulicaris Complex	*C. pulicaris*<br>*C. lupicaris clade L1*<br>*C. newsteadi clade N1*
45	28 Sept 2023	Selfkant, North Rhine-Westphalia	cattle	SBV	36.4	Pulicaris Complex	*C. punctatus*<br>*C. pulicaris*<br>*C. lupicaris clade L1*
50	28 Sept 2023	Selfkant, North Rhine-Westphalia	cattle	SBV	35.2	Obsoletus Group	*C. obsoletus clade O1/C. montanus*<br>*C. scoticus*<br>*C. dewulfi*
50	28 Sept 2023	Selfkant, North Rhine-Westphalia	cattle	SBV	35.0	Obsoletus Group	*C. obsoletus clade O1/C. montanus*<br>*C. scoticus*<br>*C. dewulfi*
50	28 Sept 2023	Selfkant, North Rhine-Westphalia	cattle	SBV	34.3	Obsoletus Group	*C. obsoletus clade O1/C. montanus*<br>*C. scoticus*<br>*C. dewulfi*
50	28 Sept 2023	Selfkant, North Rhine-Westphalia	cattle	SBV	34.9	Obsoletus Group	*C. obsoletus clade O1/C. montanus*<br>*C. scoticus*<br>*C. dewulfi*
50	28 Sept 2023	Selfkant, North Rhine-Westphalia	cattle	SBV	34.2	Obsoletus Group	*C. obsoletus clade O1/C. montanus*<br>*C. scoticus*<br>*C. dewulfi*
50	28 Sept 2023	Selfkant, North Rhine-Westphalia	cattle	SBV	35.0	Obsoletus Group	*C. obsoletus clade O1/C. montanus*<br>*C. scoticus*

Pool size, no. of biting midges	Collection date	Place of collection	Type of livestock	Virus	Cq value	Biting midge group/complex	Biting midge taxon
							C. dewulfi
50	28 Sept 2023	Selfkant, North Rhine-Westphalia	cattle	SBV	33.9	Obsoletus Group	*C. obsoletus clade O1/C. montanus*<br>*C. scoticus*
50	28 Sept 2023	Selfkant, North Rhine-Westphalia	cattle	SBV	34.4	Obsoletus Group	*C. obsoletus clade O1/C. montanus*<br>*C. scoticus*
50	28 Sept 2023	Selfkant, North Rhine-Westphalia	cattle	SBV	34.5	Obsoletus Group	*C. obsoletus clade O1/C. montanus*<br>*C. scoticus*<br>*C. dewulfi*
50	28 Sept 2023	Selfkant, North Rhine-Westphalia	cattle	SBV	35.2	Obsoletus Group	*C. obsoletus clade O1/C. montanus*<br>*C. scoticus*<br>*C. dewulfi*
50	28 Sept 2023	Selfkant, North Rhine-Westphalia	cattle	SBV	34.9	Obsoletus Group	*C. obsoletus clade O1/C. montanus*<br>*C. scoticus*<br>*C. dewulfi*
50	28 Sept 2023	Selfkant, North Rhine-Westphalia	cattle	SBV	34.5	Obsoletus Group	*C. obsoletus clade O1/C. montanus*<br>*C. scoticus*<br>*C. dewulfi*
50	28 Sept 2023	Selfkant, North Rhine-Westphalia	cattle	SBV	34.2	Obsoletus Group	*C. obsoletus clade O1/C. montanus*<br>*C. scoticus*<br>*C. dewulfi*
50	28 Sept 2023	Selfkant, North Rhine-Westphalia	cattle	SBV	34.4	Obsoletus Group	*C. obsoletus clade O1/C. montanus*<br>*C. scoticus*<br>*C. dewulfi*
50	28 Sept 2023	Selfkant, North Rhine-Westphalia	cattle	SBV	34.0	Obsoletus Group	*C. obsoletus clade O1/C. montanus*<br>*C. scoticus*<br>*C. dewulfi*
50	28 Sept 2023	Selfkant, North Rhine-Westphalia	cattle	SBV	28.5	Obsoletus Group	*C. obsoletus clade O1/C. montanus*<br>*C. scoticus*<br>*C. dewulfi*
50	28 Sept 2023	Selfkant, North Rhine-Westphalia	cattle	SBV	35.2	Obsoletus Group	*C. obsoletus clade O1/C. montanus*<br>*C. scoticus*<br>*C. dewulfi*
50	28 Sept 2023	Selfkant, North Rhine-Westphalia	cattle	SBV	33.3	Obsoletus Group	*C. obsoletus clade O1/C. montanus*<br>*C. scoticus*<br>*C. dewulfi*
50	28 Sept 2023	Selfkant, North Rhine-Westphalia	cattle	SBV	33.9	Obsoletus Group	*C. obsoletus clade O1/C. montanus*<br>*C. scoticus*<br>*C. dewulfi*
50	28 Sept 2023	Selfkant, North Rhine-Westphalia	cattle	SBV	33.5	Obsoletus Group	*C. obsoletus clade O1/C. montanus*<br>*C. scoticus*<br>*C. dewulfi*
50	28 Sept 2023	Selfkant, North Rhine-Westphalia	cattle	SBV	24.1	Obsoletus Group	*C. obsoletus clade O1/C. montanus*<br>*C. scoticus*<br>*C. dewulfi*
50	28 Sept 2023	Selfkant, North Rhine-Westphalia	cattle	SBV	28.3	Obsoletus Group	*C. obsoletus clade O1/C. montanus*<br>*C. scoticus*<br>*C. dewulfi*<br>*C. chiopterus*

Pool size, no. of biting midges	Collection date	Place of collection	Type of livestock	Virus	Cq value	Biting midge group/complex	Biting midge taxon
50	28 Sept 2023	Selfkant, North Rhine-Westphalia	cattle	SBV	36.6	Obsoletus Group	*C. obsoletus clade O1/C. montanus*<br>*C. scoticus*<br>*C. dewulfi*
50	28 Sept 2023	Selfkant, North Rhine-Westphalia	cattle	SBV	35.8	Obsoletus Group	*C. obsoletus clade O1/C. montanus*<br>*C. scoticus*<br>*C. dewulfi*
50	28 Sept 2023	Selfkant, North Rhine-Westphalia	cattle	SBV	35.2	Obsoletus Group	*C. obsoletus clade O1/C. montanus*<br>*C. scoticus*<br>*C. dewulfi*
50	28 Sept 2023	Selfkant, North Rhine-Westphalia	cattle	SBV	36.9	Obsoletus Group	*C. obsoletus clade O1/C. montanus*<br>*C. scoticus*<br>*C. dewulfi*
50	28 Sept 2023	Selfkant, North Rhine-Westphalia	cattle	SBV	34.0	Obsoletus Group	*C. obsoletus clade O1/C. montanus*<br>*C. scoticus*<br>*C. dewulfi*
50	28 Sept 2023	Selfkant, North Rhine-Westphalia	cattle	SBV	37.5	Obsoletus Group	*C. obsoletus clade O1/C. montanus*<br>*C. scoticus*<br>*C. dewulfi*<br>*C. chiopterus*
50	28 Sept 2023	Selfkant, North Rhine-Westphalia	cattle	SBV	31.7	Obsoletus Group	*C. obsoletus clade O1/C. montanus*<br>*C. scoticus*<br>*C. dewulfi*
50	28 Sept 2023	Selfkant, North Rhine-Westphalia	cattle	SBV	33.9	Obsoletus Group	*C. obsoletus clade O1/C. montanus*<br>*C. scoticus*<br>*C. dewulfi*
50	03 Oct 2023	Nordhorn, Lower Saxony	cattle	SBV	34.1	Obsoletus Group	*C. obsoletus clade O1/C. montanus*<br>*C. scoticus*
50	03 Oct 2023	Nordhorn, Lower Saxony	cattle	SBV	38.3	Obsoletus Group	*C. obsoletus clade O1/C. montanus*<br>*C. scoticus*<br>*C. dewulfi*
50	03 Oct 2023	Nordhorn, Lower Saxony	cattle	SBV	32.6	Obsoletus Group	*C. obsoletus clade O1/C. montanus*<br>*C. scoticus*
50	03 Oct 2023	Nordhorn, Lower Saxony	cattle	SBV	36.1	Obsoletus Group	*C. obsoletus clade O1/C. montanus*<br>*C. scoticus*<br>*C. dewulfi*
50	03 Oct 2023	Nordhorn, Lower Saxony	cattle	SBV	34.6	Obsoletus Group	*C. obsoletus clade O1/C. montanus*<br>*C. scoticus*
50	03 Oct 2023	Nordhorn, Lower Saxony	cattle	SBV	36.5	Obsoletus Group	*C. obsoletus clade O1/C. montanus*<br>*C. scoticus*
50	04 Oct 2023	Nordhorn, Lower Saxony	cattle	SBV	39.6	Obsoletus Group	*C. obsoletus clade O1/C. montanus*<br>*C. scoticus*<br>*C. dewulfi*
50	04 Oct 2023	Nordhorn, Lower Saxony	cattle	SBV	32.7	Obsoletus Group	*C. obsoletus clade O1/C. montanus*<br>*C. scoticus*<br>*C. dewulfi*
49	04 Oct 2023	Nordhorn, Lower Saxony	cattle	SBV	37.1	Obsoletus Group	*C. obsoletus clade O1/C. montanus*<br>*C. scoticus*<br>*C. dewulfi*

Pool size, no. of biting midges	Collection date	Place of collection	Type of livestock	Virus	Cq value	Biting midge group/complex	Biting midge taxon
13	04 Oct 2023	Nordhorn, Lower Saxony	cattle	SBV	24.7	Obsoletus Group	*C. obsoletus clade O1/C. montanus C. scoticus*
50	05 Oct 2023	Nordhorn, Lower Saxony	cattle	SBV	33.5	Obsoletus Group	*C. obsoletus clade O1/C. montanus C. scoticus*
50	05 Oct 2023	Nordhorn, Lower Saxony	cattle	SBV	31.0	Obsoletus Group	*C. obsoletus clade O1/C. montanus C. scoticus C. dewulfi*
50	05 Oct 2023	Nordhorn, Lower Saxony	cattle	SBV	30.3	Obsoletus Group	*C. obsoletus clade O1/C. montanus C. scoticus C. chiopterus*
50	01 Oct 2023	Nordhorn, Lower Saxony	cattle	SBV	39.9	Obsoletus Group	*C. obsoletus clade O1/C. montanus C. scoticus C. dewulfi*
50	01 Oct 2023	Nordhorn, Lower Saxony	cattle	SBV	39.4	Obsoletus Group	*C. obsoletus clade O1/C. montanus C. scoticusC. chiopterus*
50	01 Oct 2023	Nordhorn, Lower Saxony	cattle	SBV	40.4	Obsoletus Group	*C. obsoletus clade O1/C. montanus C. scoticus C. dewulfi C. chiopterus*
50	01 Oct 2023	Nordhorn, Lower Saxony	cattle	SBV	39.1	Obsoletus Group	*C. obsoletus clade O1/C. montanus C. scoticus C. dewulfi*
50	01 Oct 2023	Nordhorn, Lower Saxony	cattle	SBV	40.5	Obsoletus Group	*C. obsoletus clade O1/C. montanus C. scoticus C. dewulfi*
50	01 Oct 2023	Nordhorn, Lower Saxony	cattle	SBV	40.2	Obsoletus Group	*C. obsoletus clade O1/C. montanus C. scoticus C. dewulfi C. chiopterus*
50	01 Oct 2023	Nordhorn, Lower Saxony	cattle	SBV	39.2	Obsoletus Group	*C. obsoletus clade O1/C. montanus C. scoticus C. dewulfi*
50	01 Oct 2023	Nordhorn, Lower Saxony	cattle	SBV	32.7	Obsoletus Group	*C. obsoletus clade O1/C. montanus C. scoticus C. chiopterus*
50	01 Oct 2023	Nordhorn, Lower Saxony	cattle	SBV	40.4	Obsoletus Group	*C. obsoletus clade O1/C. montanus C. scoticus C. dewulfi C. chiopterus*
50	01 Oct 2023	Nordhorn, Lower Saxony	cattle	SBV	34.4	Obsoletus Group	*C. obsoletus clade O1/C. montanus C. scoticus*
50	03 Nov 2023	Nordhorn, Lower Saxony	cattle	SBV	35.4	Obsoletus Group	*C. obsoletus clade O1/C. montanus C. scoticus*

*Cq, quantification cycle.
